# Printable Bifocal Microlenses from Ferroelectric Nematic Liquid Crystal Droplets

**DOI:** 10.1021/acsami.6c06633

**Published:** 2026-06-08

**Authors:** Manisha Talwar, Zakaria Siddiquee, Antal Jákli

**Affiliations:** † Department of Physics, 4229 Kent State University, Kent, Ohio 44242, United States; ‡ Advanced Materials and Liquid Crystal Institute, Kent State University, Kent, Ohio 44242, United States

**Keywords:** ferroelectric nematic liquid crystals, bifocal microlens arrays, self-assembled liquid crystal lenses, optical properties of liquid crystals, birefringent lenses

## Abstract

Multifocal lenses are essential for enhancing stereoscopic image display and increasing the depth of field in optical devices. Traditional liquid crystal lenses face challenges in achieving planar alignment at both interfaces, which limits their use for multifocal lenses. Here, we demonstrate printable bifocal microlens arrays formed by submillimeter plano-convex droplets of ferroelectric nematic liquid crystals deposited on rubbed polymer-coated glass substrates, promoting uniform planar alignment. These droplets exhibit two polarization-dependent focal lengths, corresponding to the extraordinary and ordinary refractive indices of the material. Computer simulations corroborate the observed director structures and optical behaviors across the nematic, antiferroelectric smectic, and ferroelectric nematic phases. Our findings reveal a straightforward method for self-assembled bifocal lenses, offering potential applications in autostereoscopic displays and devices requiring an enhanced depth of field without complex fabrication processes.

## Graphical abstract

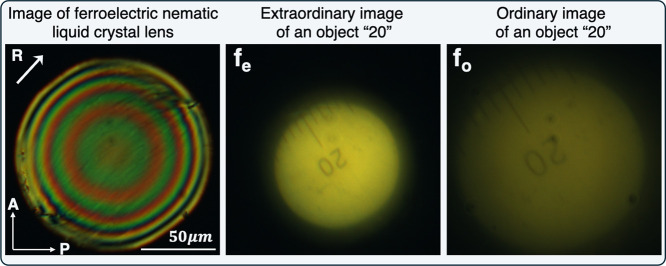

## 1 Introduction

Liquid crystals (LCs) are mostly studied and used in thin films where the material is sandwiched between two parallel glass substrates that provide uniform director alignment. This geometry simplifies the measurements of the physical, especially the optical properties of the LCs. However, their preparation requires a complicated procedure with the deposition of a uniform, nanometer-thick alignment layer, keeping a uniform, micrometer-scale film thickness and inserting the LC without air bubbles. For this reason, the first LC studies at the end of the 19th century by Otto Lehmann were carried out in sessile droplet forms.^1^ The problem with such droplets is the complicated director structure (including defect lines and walls), even in the simplest nematic phase.^2^ Additionally, because of the varying director structure and film thickness, the textures seen under polarized microscopes are also difficult to interpret.

On the other hand, as sessile droplets have plano-convex lens shape, they are potentially useful for tunable, multifocal lenses that currently have a great demand in augmented reality (AR)/virtual reality (VR) technology to overcome the vergence-accommodation conflict and in displaying stereoscopic images with increased depth of field (DOF).^3^ Self-assembly of birefringent lenses that can be useful for stereoscopy and increased DOF requires planar alignment (LC director is parallel to the surface) on both the LC-glass and LC-air interfaces. Although there are liquid crystals that align parallel to the air interface, such as azoxyanisole (PAA)^4^ and some dimers,^5^ most calamitic LCs, such as cyanobiphenyls, align perpendicular (so-called homeotropic alignment) at the air interface.^6^ For this reason, the realization of these LC lenses currently requires relatively complicated techniques such as sandwiching them between solid lenses,^7,8^ making bilayer LC structures,^9^ creating hole electrodes,^10^ and using microfluidic technology,^11,12^ two-step photolithography, and thermal reflow.^13^

A solution of this problem is offered by the recently discovered ferroelectric nematic (N_F_) liquid crystals^14,15^ that all tend to align parallel to insulating surfaces^16,17^ such as to LC-air interface.^18^ This is because a large depolarization field $E_{dep}=-\frac{P_{o}\sin\;\theta }{\varepsilon_{o}\varepsilon }$ appears when the spontaneous polarization (*P*_o_∼6 μC/cm^2^) makes an angle θ > 0 with respect to the interface between an insulating layer and the N_F_ material with ε_o_ε dielectric permittivity, where ε_o_ = 8.85 × 10^–12^ C^2^ m^–2^ N^–1^ is the permittivity of the free space. Importantly, this depolarization field forces tangential alignment at the air–solid substrate boundary, which is different from the alignment of planar paraelectric nematics that have radial director structure, leading to defect in the middle of the droplet. Plano-convex shape N_F_ lenses so far have been studied on insulating substrates in electric fields^18–20^ and on solid ferroelectric surfaces^21,22^ showing instabilities and peculiar dynamical behavior. It has been also demonstrated that suspending chiral ferroelectric nematic (N_F_*) liquid crystal in submillimeter size transmission electron microscopy (TEM) grids leads to biconvex lenses that provide tunable focal length when they are polymer stabilized in the isotropic phase.^23,24^ Although these lenses form spontaneously, they require TEM grids, thus their size and geometry are limited.

To overcome this constraint and to achieve printable birefringent lenses, here, we study LC droplets in their nematic, antiferroelectric smectic and ferroelectric nematic phases deposited on glass substrates by a microplotter. Specifically, we study the shape, director structure, and optical properties of submillimeter-size plano-convex droplets of a room-temperature ferroelectric nematic mixture FNLC 919 from Merck on glasses coated by rubbed polymers that promote uniform planar alignment. Compared to previously reported approaches based on TEM grids, the microplotter technique enables direct and scalable printing of liquid crystal droplets with controlled size, spacing, and geometry. In addition, the printing process provides flexibility in arranging the droplets into ordered arrays, which are essential for practical applications. Here, we find that the droplets may form spherical cap or obround shapes that have two light-polarization-dependent focal lengths determined by the temperature-dependent extraordinary and ordinary refractive indices of the material. Measuring the focal lengths and the curvature radii, we determine the apparent refractive indices and birefringence and carry out computer simulations that explain the shapes, director structure, and optical properties of the droplets in all three LC phases of the material. These self-assembled bifocal lens arrays, where the two focal lengths arise from the difference between the ordinary and extraordinary refractive indices of liquid crystals, may be useful in displaying stereoscopic images with an increased depth of field.

## 2 Materials and Methods

FNLC-919 was provided to us by Merck Electronics, KGaA, Darmstadt, Germany. Its phase sequence on cooling was reported as I-80 °C–N-44 °C–N_*x*_-32 °C–N_F_-8 °C–Cr, where N_*x*_ is the tentative identifier for the phase intermediate between the conventional N and ferroelectric N_F_ phases. FNLC-919 has been studied by various groups describing their alignment properties and birefringence,^25^ its ability to form free-standing filaments,^26^ and its use in electrically tunable lenses and reflectors.^23,27^ Recently, the temperature dependences of the key material parameters such as ferroelectric polarization and viscosity were characterized in all mesophases, while the orientational elastic constants, dielectric permittivity, and diamagnetic anisotropy were determined only in the N and N_X_ phases.^28^ It was also shown^28^ that the nanostructure of the N_X_ phase is identical to the antiferroelectric smectic SmZ_A_ phase^29^ existing above the N_F_ phase of the prototype ferroelectric nematic material DIO.^15^

Deposition of FNLC-919 droplets were carried out by the following procedures. First, flat glass substrates, served as base plates of the sessile droplets, were spin-coated by a polyimide SE2170 and rubbed uniformly by a velvet cloth 10 times in one direction, by applying 490 Pa pressure providing uniform planar alignment with nematic director along the rubbing (R) direction. A few microliters of FNLC-919 material were suctioned in a 10 μm diameter glass needle through capillary action. The needle was attached to an AC current-driven piezoelectric element that induces vibration producing a resonant pumping action of the needle that expels fluid enabling the deposition of droplets. Using a SonoPlotMicroplotter II, the position of the dispenser was controlled laterally with 5 μm precision by Sonoguide software, making it possible to print droplet arrays with parameters programmed by SonoDraw software directly onto glass substrates as shown in Figure 1a.

**Figure 1:**
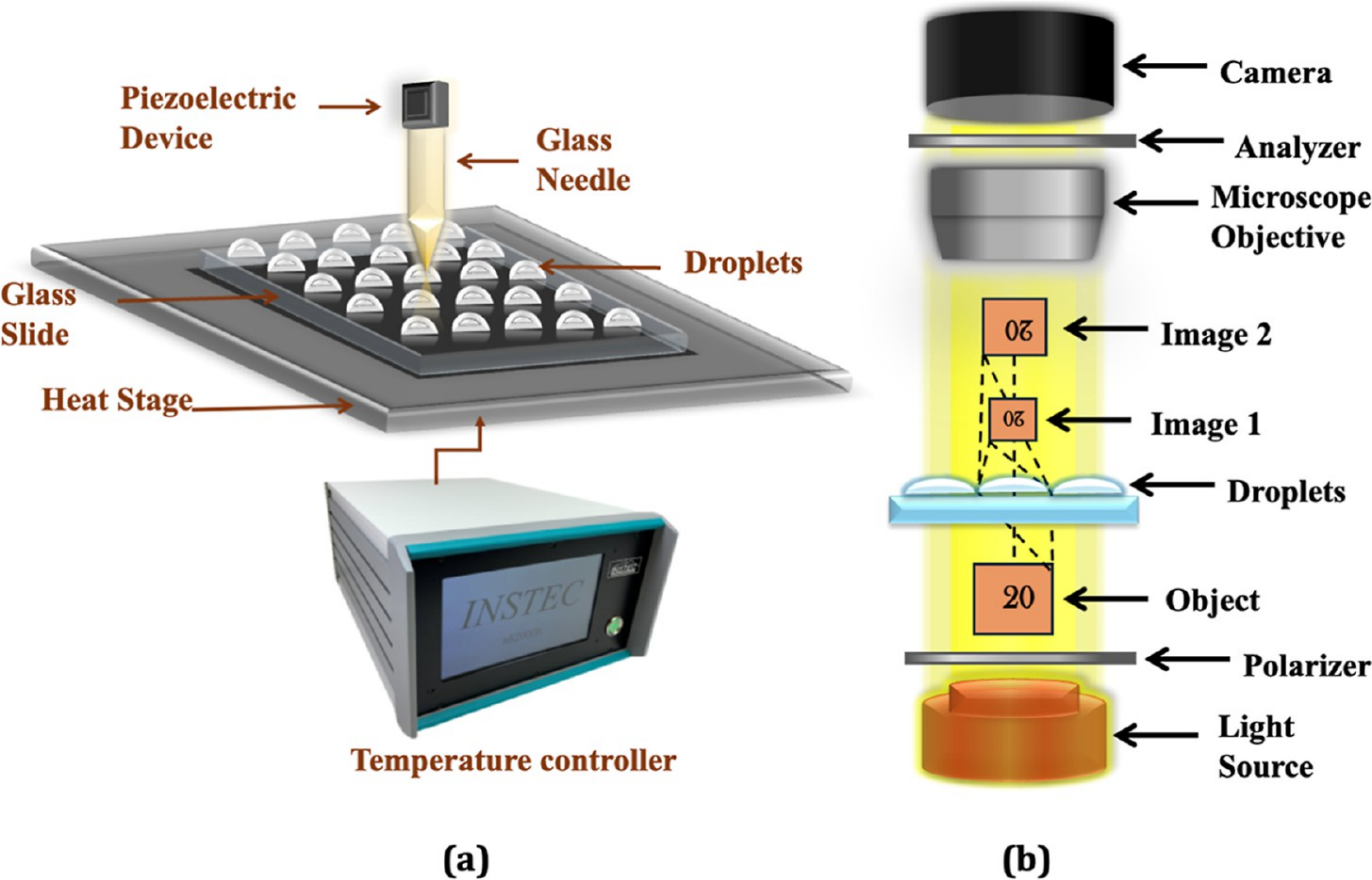
Schematics of the experimental set up for droplet deposition and for its characterization. (a) Illustration of the droplet deposition on the glass substrate using a microplotter and (b) polarized optical microscopy (POM) setup to visualize the droplets, the object (a number **20**), and its inverted images for measuring the focal distances.

After printing, the shape and texture of the printed FNLC919 droplets were studied with an Olympus BX60 polarizing optical microscope (POM).

To test the imaging ability of the plano-convex lens-shaped droplets, a microscope calibration slide was mounted on a micropositioner and carefully adjusted until an inverted image of the number **20** of the calibration slide became visible on the other side of the microlens array. All images were captured using a Pixelink PL-D752CU camera, attached to the microscope. The experimental setup used to observe the microlenses is shown in Figure 1b.

Side views of droplets were captured using a Navitar zoom lens coupled with an AmScope camera. The zoom lens provided a maximum of 5-fold magnification. For further magnification, a 20Χ microscope objective lens was placed in front of it.

## 3 Results and Discussion

The optical focusing properties of droplets deposited at room temperature on a uniformly rubbed SE 2170 substrate are illustrated in Figure 2 using only one polarizer or analyzer, and Figure 3 shows when the sample is between crossed polarizers.

**Figure 2:**
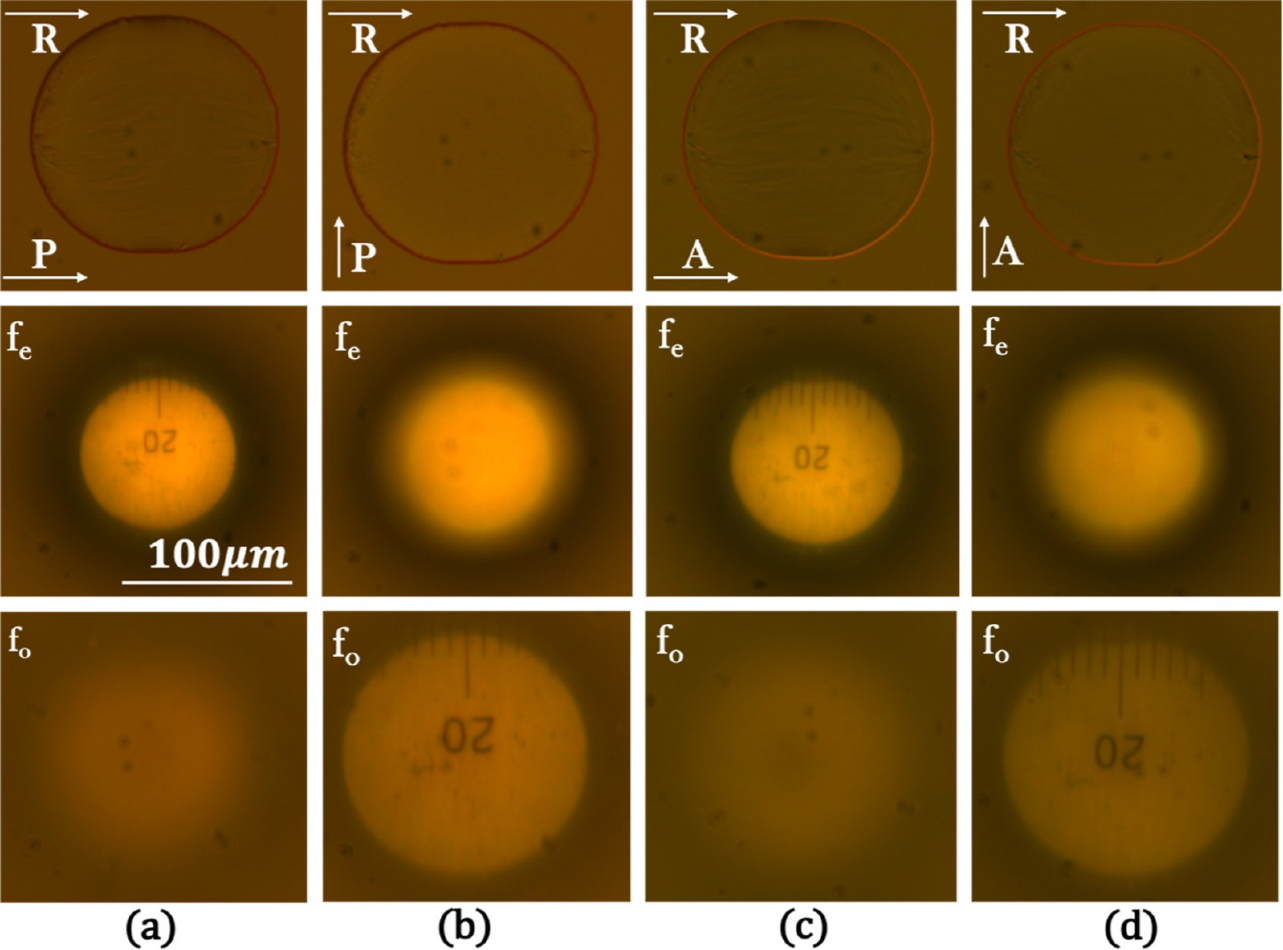
Illustration of the optical focusing properties at room temperature of an FNLC 919 droplet deposited at room temperature on a horizontally rubbed SE 2170 substrate coated on a glass plate using only one polarizer or analyzer. Top row: image of the droplet; middle row: inverted image of an object “20”at focal length *f*_e_; and bottom row: inverted image of an object “20”at focal length *f*_*o*_. (a) there is a polarizer in front of the lens-shaped droplet along the rubbing direction; (b) there is a polarizer in front of the lens-shaped droplet perpendicular to the rubbing direction; (c) there is an analyzer behind of the lens-shaped droplet along the rubbing direction; and (d) there is an analyzer behind of the lens-shaped droplet perpendicular to the rubbing direction.

**Figure 3:**
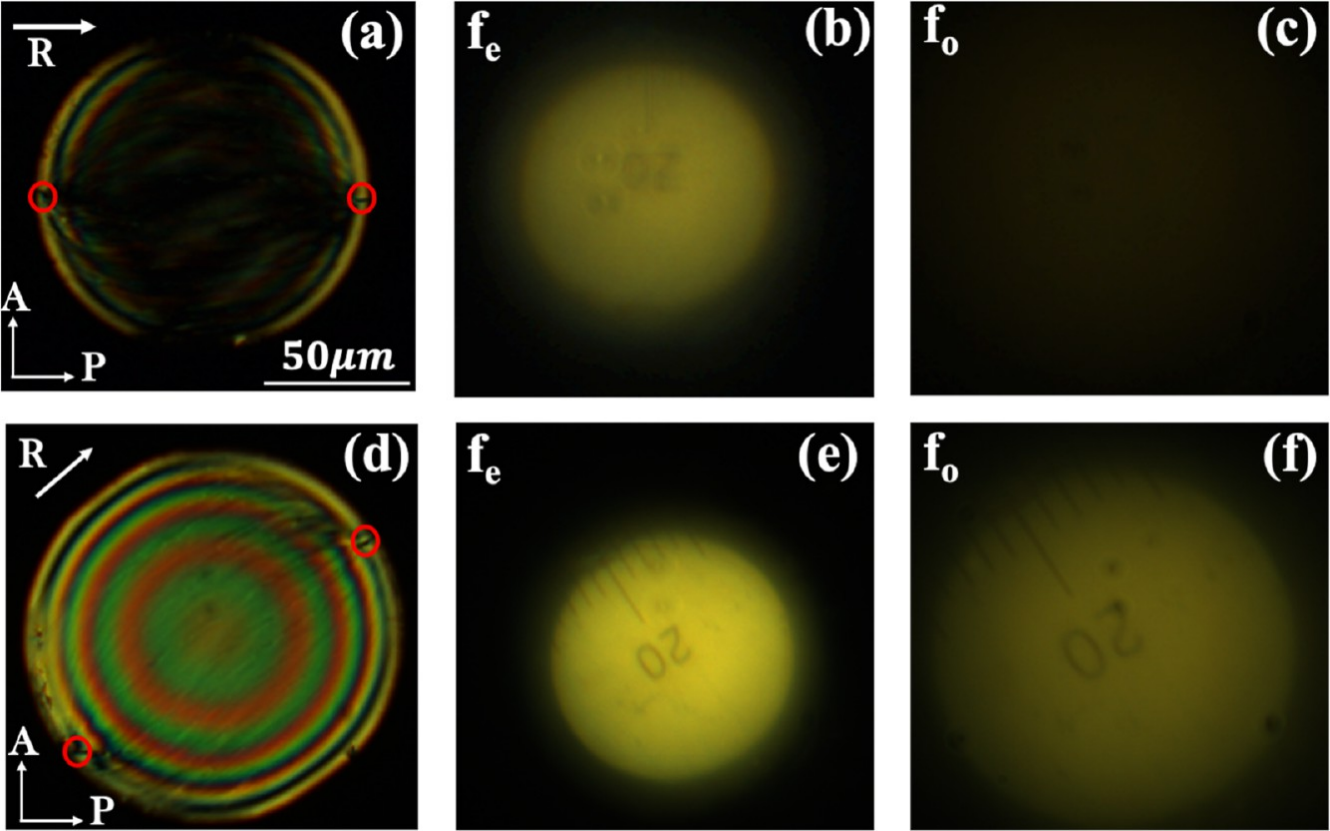
Illustration of the optical focusing properties at room temperature of an FNLC 919 droplet deposited at room temperature on a horizontally rubbed SE 2170 substrate coated on a glass plate when the droplet is between crossed polarizers. (a–c) the rubbing direction is parallel to the polarizer and (d–f) the rubbing direction is at 45° with respect to the polarizer. (a and d) show the images of the droplets; (b and e) show the images of the droplet at distance *f*_e_; and (c and f) show the images of the droplet at distance *f*_o_. Red circles show the locations of 1/2 defects in the droplet.

The top row of Figure 2 shows POM images of a droplet at room temperature. The middle and bottom rows show inverted images of an object “20” at focal lengths determined by measuring the object distance (*l*_o_) and image distance (*l*_*i*_) from the equation $f=\frac{l_{o}\times l_{i}}{{l_{o}+l}_{i}}$ . The shorter focal length is named *f*_e_ and the longer one *f*_o_. When there is a polarizer in front of the lens-shaped droplet along the rubbing direction (Figure 2a) only the image at *f*_e_ is clearly visible, while the image at *f*_o_ is blurred. When the polarizer in front of the lens-shaped droplet is perpendicular to the rubbing direction (Figure 2b) only the image at *f*_o_ is focused, while the image at *f*_e_ is blurred. The same is true for situations when there is no polarizer but only an analyzer behind the droplet: when the analyzer is along (perpendicular to) the rubbing direction, then the image at *f*_e_ (*f*_*o*_)­is focused and the image at *f*_o_ (*f*_e_)­is blurred (Figure 2c,d).

Figure 3 shows the droplet and the images focused at *f*_e_ and *f*_o_ between crossed polarizers. Top row (Figure 3a–c) shows the situation when the rubbing direction is parallel to the polarizer. In this case, the polarized optical microscopy image of the droplet seen in Figure 3a is dark everywhere except at the edge indicating uniform alignment in most of the droplet. Also, no image can be seen at *f*_o_ (see Figure 3c). When the rubbing direction makes some angle (e.g., 45° as shown in the bottom row of Figure 3) with respect to the polarizer or analyzer, the images at both *f*_e_ and *f*_o_ are focused. This is because the light propagating within the LC splits between ordinary and extraordinary beams that experience extraordinary refractive index *n*_e_ and ordinary index *n*_o_ when the electric vector is parallel and perpendicular to the director, respectively.^30^

The temperature dependences of the droplet geometric parameters (height *h* and curvature radius *R*), the measured focal lengths, the calculated refractive indices, and effective birefringence values of the same FNLC 919 droplet, deposited on a SE 2170-coated glass plate at room temperature and heated at a 0.5 °C/min rate up to the isotropic phase, are plotted in Figure 4. Figure 4a shows the temperature-dependent contact angle θ and central height *h*. These values and the diameters 2*a* were measured from the side views (see inset to Figure 4a). The contact angle θ of droplet was determined by the side view of the droplet profile in two ways. First, from the measured height *h*, base radius (*a*), and calculated curvature radius $R\approx \frac{a^{2}}{2h}$ as $\sin\theta =\frac{a}{R}$ , which is valid for shallow spherical caps. Second, by fitting a tangent to the liquid–air interface at the three-phase contact line using ImageJ. One can see that the contact angle is θ ≈ 18° in the N_F_ and N_X_ phases, then drops below 16° in the N phase. An especially large decrease of the height and contact angle is seen in the middle of the N phase between 68 and 71 °C. To emphasize it, this range is shaded by gray. It is also remarkable that above 75 °C the height and contact angle are the same as in the isotropic phase.

**Figure 4:**
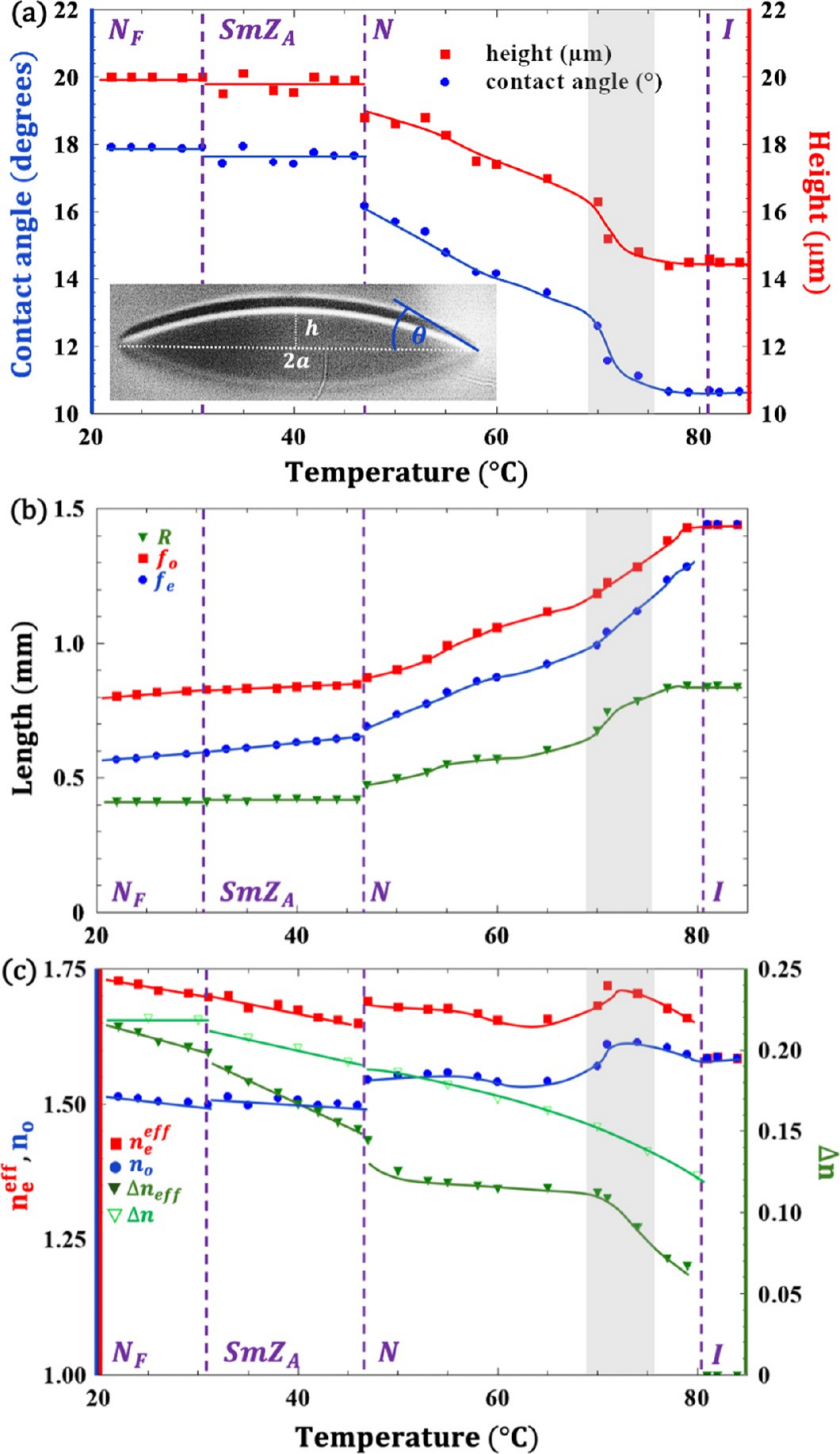
Temperature dependences of the droplet geometric parameters, the measured focal lengths, the calculated refractive indices, and effective birefringence values of an FNLC 919 droplet, deposited on a SE 2170-coated glass plate at room temperature and heated at a 0.5 °C/min rate up to the isotropic phase. (a) Height *h* (solid red squares, right axis) and contact angle θ (solid blue circles, left axis). Inset shows the side view of the droplet (top half) and its mirror image (bottom part) with the indication of the heigh *h*, contact angle θ, and the diameter 2*a*. (b) Curvature radius *R* (solid green triangles) and measured focal lengths *f*_e_ (solid blue circles) and *f*_o_ (solid red squares). (c) Calculated refractive indices *n*_e_^eff^ (solid red squares), *n*_o_ (solid blue circles) and the effective birefringence Δ*n*_eff_ = *n*_e_^eff^ – *n*_o_ (solid green triangles). Light green open triangles show the birefringence values measured on planar aligned films by Yu et al.^25^

Temperature dependence of the curvature radius $R=\frac{a^{2}}{2h}$ , determined from the temperature-dependent side views (inset to Figure 4a), is shown in Figure 4b. The *R* values were also determined from fitting the side view images by Tracker software and found to match with those achieved from the *h* and *a* measurements within an uncertainty of ±0.05 mm. Figure 4b also shows the temperature dependence of the focal lengths *f*_e_ and *f*_o_ with an estimated uncertainty of ± 5 μm. As seen, *f*_o_ increases from ≈0.8 mm at 25 °C to ≈1.4 mm at the transition to the isotropic phase at 81 °C, while *f*_e_ increases from ≈0.57 mm at 25 °C to ≈1.3 mm at 81 °C. For both cases, the increase in the N_F_ and N_X_ range is very small and linear without any observable jump at the N_F_–N_X_ transition. The slope of the increase is much larger in the N phase with a visible modulation in the 68–76 °C range where the height and the contact angles showed a large decrease as shown in Figure 4a.

Using lens maker’s equation, $f=\frac{R}{n-1}$ valid for thin lenses such as our FNLC 919 droplets, from the measured curvature radius *R* and the focal lengths *f*_e_ and *f*_o_ values, one can calculate the apparent extraordinary and ordinary refractive indices as $n_{e}^{eff}=1+\frac{f_{e}}{R}$ and $n_{o}=1+\frac{f_{o}}{R}$ with an estimated error of ±0.005. The temperature dependences of *n*_e_^eff^ and *n*_o_ and the effective birefringence Δ*n*_eff_ = *n*_e_^eff^ – *n*_o_ are shown in Figure 4c. The ordinary refractive index increases from 1.51 at 25 °C to 1.61 at 70 °C, while the apparent extraordinary index decreases from 1.73 at 25 °C to 1.65 at 47 °C. In the N phase, *n*_e_^eff^ (*T*) is nonmonotonous showing a maximum of 1.72 at 70 °C. The effective birefringence at 25 °C is Δ*n*_eff_ ≈ 0.22, which is practically the same as measured by Yu et al.^25^ in planar aligned films. Together with the uniformly dark image of the droplet seen between crossed polarizers parallel to the rubbing direction (see Figure 3a), this shows almost uniform planar alignment of the droplet. On heating, however, Δ*n*_eff_ measured in the droplet becomes smaller than the literature value of Δ*n* shown by light green open triangles. This indicates that on heating to the N phase, the average alignment deviates from the planar. As the base plate has planar alignment coating, this means that the air-LC alignment becomes increasingly tilted on heating. A steep decrease of Δ*n*_eff_ above 68 °C, indicates a transition to homeotropic alignment, which actually is the usual situation for conventional nematic liquid crystals at the air-LC interface.

To verify this hypothesis, we have monitored the shape and texture of the droplets upon heating from room temperature. Snapshots at selected temperatures both upon heating and cooling are shown in Figure 5. The base of the droplet is circular in the N_F_ and N_X_ phases and at the low-temperature range of the N phase, but a considerable elongation along the rubbing direction starts at around 65 °C when the number of interference rings drops from 5 to 2, then to 1 by 73 °C. On further heating, the texture becomes gradually dark due to transition to the homeotropic alignment of the entire droplet at about 76 °C before it would transition to the isotropic phase at 81 °C. On cooling, the homeotropic domains are partially seen even at 73 °C, especially at the thinnest edge of the droplet. Apart from a wrinkling of the interference fringes seen at 38 °C and at 47 °C, no defect walls are seen in this droplet. Interestingly, however, the elongated obround shape persists even at room temperature and the number of interference rings (i.e., the height and contact angle) on cooling is less in the SmZ_A_ and N_F_ phases than upon heating.

**Figure 5:**
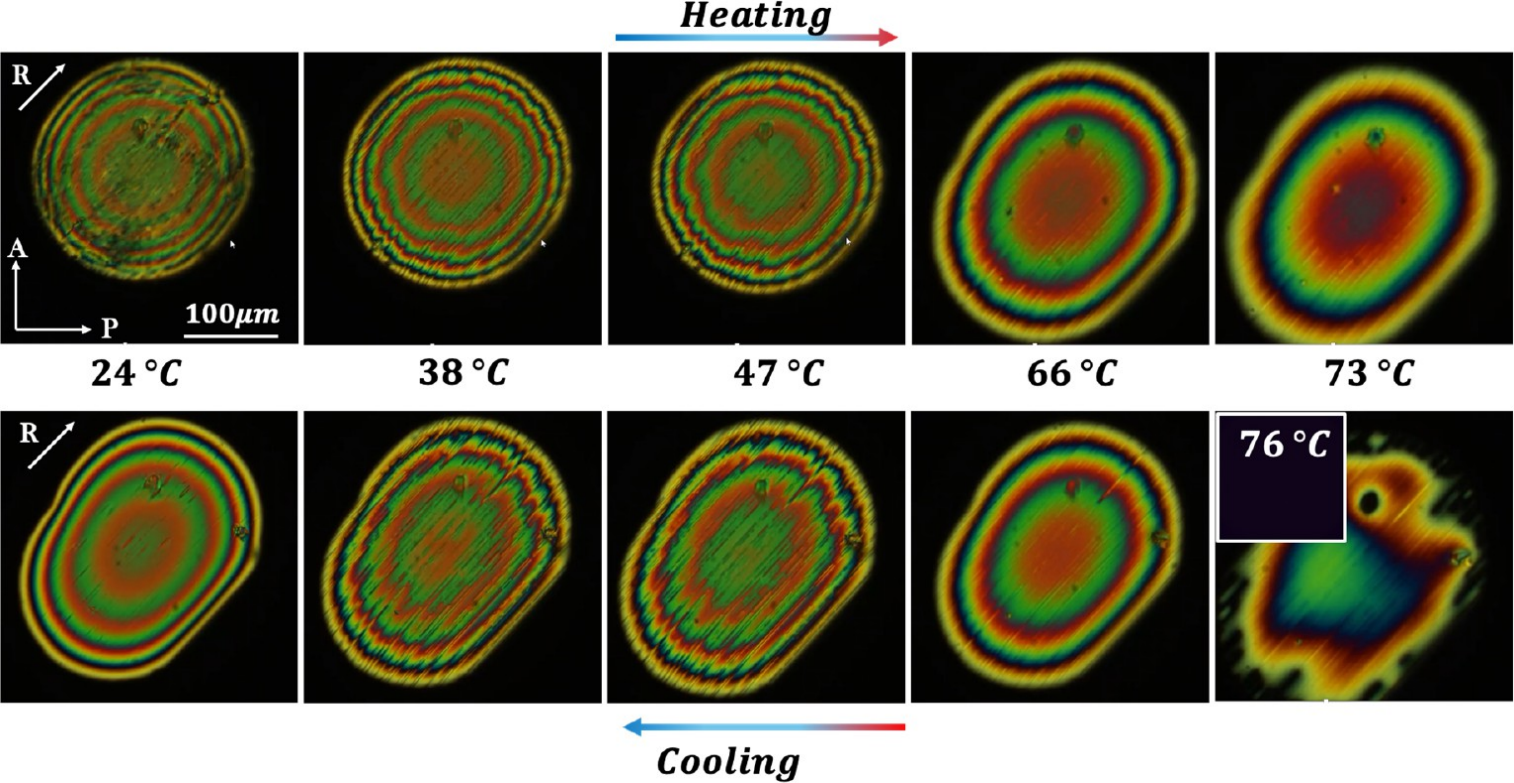
Top view of the POM images of an FNLC 919 droplet deposited on a SE 2170-coated glass plate at room temperature and heated at a 0.5 °C/min rate to the isotropic phase. The white arrow labeled by R shows the rubbing direction of the SE2170 substrate, and vectors labeled A and P show the direction of the analyzer and polarizer. The inset to the texture at 73 °C on cooling shows the uniformly homeotropic texture seen at 76 °C.

These observations agree with the results shown in Figure 4 that indicated a gradual decrease of the birefringence upon heating to the N phase, especially above 68 °C, and then leading to homeotropic alignment in the thin periphery of the droplets even at the solid substrate to reduce the Frank elastic energy.^31^ This is the result of the lack of ferroelectric polarization, thus the lack of depolarization field, which would constrain the polarization (thus the director) parallel to the interfaces and the temperature-dependent surface anchoring that prefers homeotropic anchoring at the air interface for most calamitic nematic liquid crystals.

Additionally, we also find that the base shape becomes elongated along the rubbing direction where previous measurements showed the steep decrease of the contact angle. Looking at Figure 4b, we see that the elongation happens when the contact angle θ becomes less than 13°, i.e., when $\cos\theta =\frac{{\gamma_{s-air}-\gamma }_{s-LC}}{\gamma_{LC-air}}\geq 0.98$ . This happens for a small γ_s–LC_ when the penalty we pay upon the elongation of the droplet is small and it can be overcompensated by the decrease of the anchoring energy due to the increased area where the favored director alignment is along the rubbing direction. The fact that on cooling the obround shape persists even in the N_F_ phase is likely due to the adhesion between the surface and of the LC molecules stuck in the elongated area.

The “wrinkling” of the interference fringes in the SmZ_A_ phase observed both in heating and cooling is likely related to the layered structure of this phase.^32^ On heating, the layer spacing decreases^32^ leading to layer buckling in the form of chevron^33,34^ and “striped bookshelf”^35^ textures observed previously in ferroelectric SmC* materials. Such textures result in periodic director rotation and local change of the apparent birefringence, leading to a periodic shift of the position of the interference fringes, as illustrated in Figure 6a.

**Figure 6:**
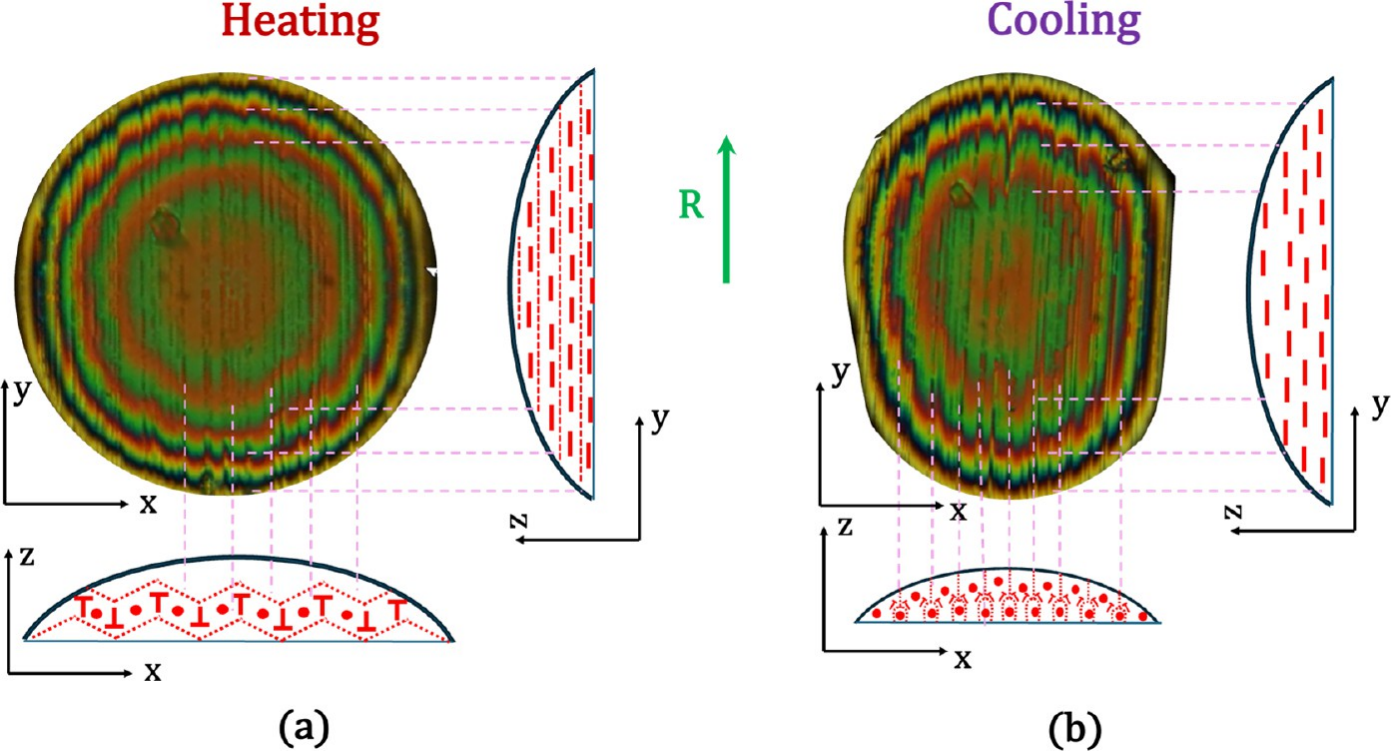
Illustration of the “wrinkle” formation of the interference rings seen in the SmZ_A_ phase on heating (a) and in cooling (b).

On cooling the layers prefer to be perpendicular to the substrate (bookshelf alignment).^32^ In this case, the layer spacing increases on cooling, so no layer undulations should appear. On the other hand, due to a temperature gradient in the droplets that are heated from the bottom, the number of layers is larger at the bottom of the sample, leading to periodic formation of dislocations as shown in Figure 6b. Again, in the areas of the disclinations the effective birefringence is smaller, leading to the wrinkling of the interference fringes. Such a gradient was analyzed by Máthé et al.^18^

As the elongated shape of lenses is not desirable for optical focusing, printed samples studied later were not heated over 60 °C to keep the circular shape of the base. Studies of a large number of droplets deposited with virtually the same condition at room temperature and heated/cooled at similar rates showed slight variation of the size and the exact textures. In spite of these, there are common features that are characteristic to the phase and cooling/heating conditions.

Typical POM textures with rubbing directions both along and at 45° between the crossed polarizers, together with their simulated director structures and simulated POM textures are shown on heating and cooling in Figures 7 and 8, respectively.

**Figure 7:**
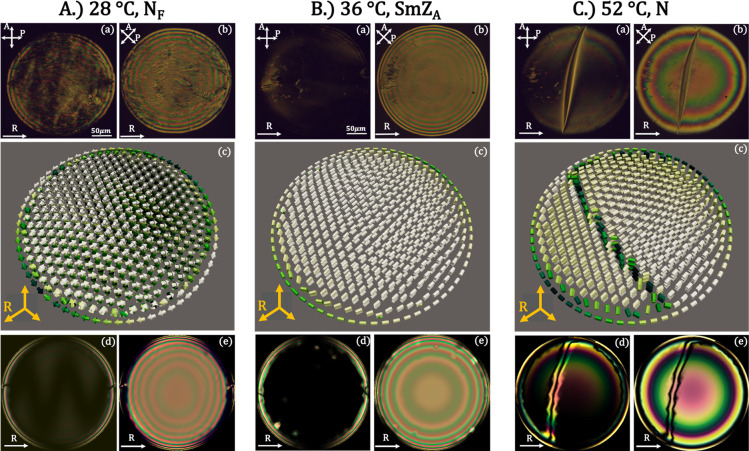
Typical POM textures with rubbing directions along and at 45° between the crossed polarizers, together with their simulated director structures and simulated POM textures measured and simulated on **heating** at a 0.5 °C/min rate from room temperature up to 52 °C. A: 28 °C in the N_F_ phase; B: 36 °C in the SmZ_A_ phase; and C: 52 °C in the N phase. (a) observed POM textures with crossed polarizers along the rubbing direction; (b) observed POM textures with crossed polarizers at 45 °C with the rubbing direction; (c) 3D rendering of simulated director structures; (d) simulated POM textures with crossed polarizers along the rubbing direction; and (e) simulated POM textures with crossed polarizers at 45 °C with the rubbing direction. The lighter the color of the columns, the closer is the director to the rubbing direction. Dark green columns indicate director perpendicular to the rubbing.

**Figure 8:**
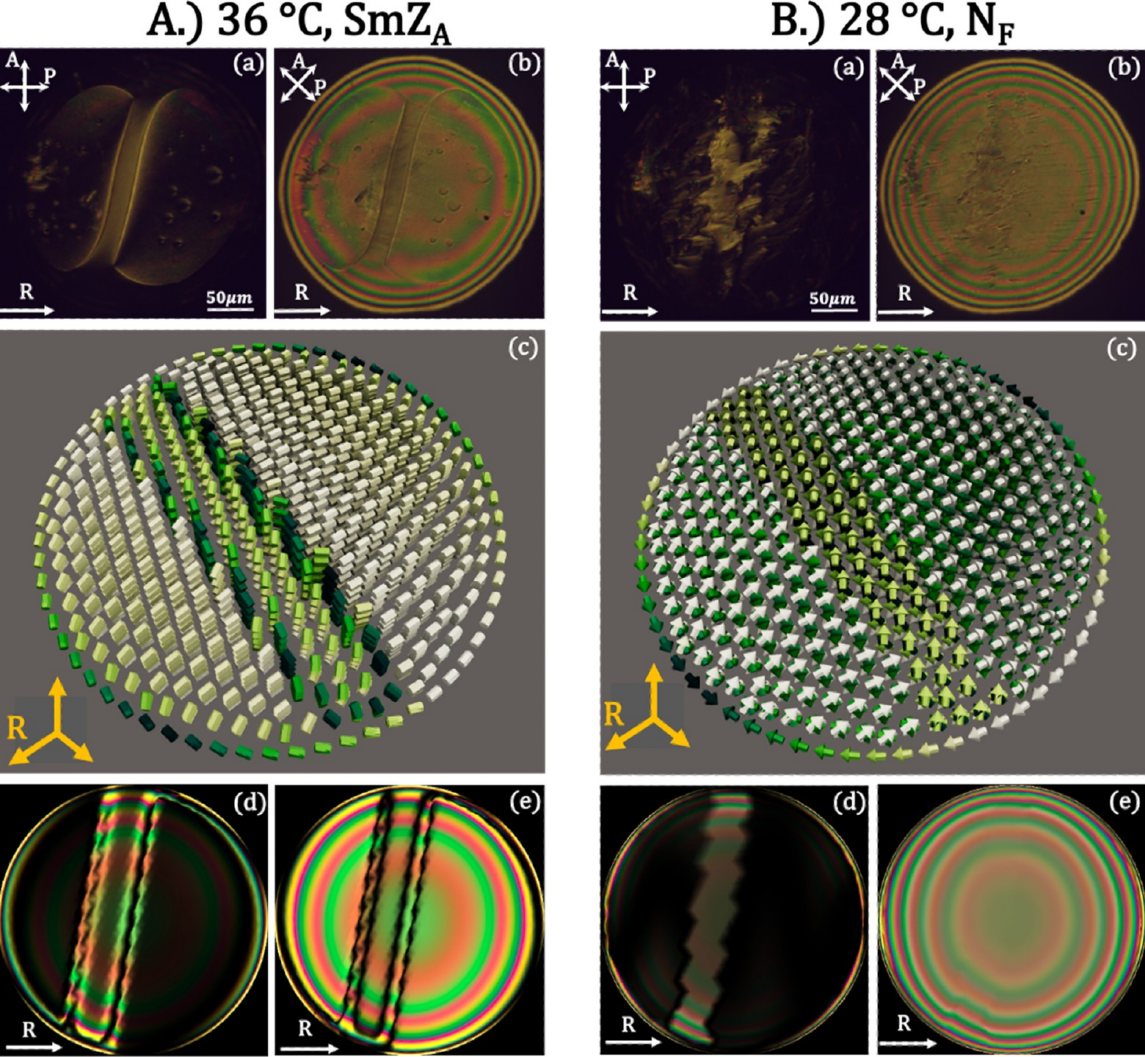
Typical POM textures with rubbing directions along and at 45° between the crossed polarizers, together with their simulated director structures and simulated POM textures measured and simulated on **cooling** at a 0.5 °C/min rate from 52 °C to room temperature. (A) 36 °C in the SmZ_A_ phase and (B) 28 °C in the N_F_ phase. (a) observed POM textures with crossed polarizers along the rubbing direction; (b) observed POM textures with crossed polarizers at 45 °C with the rubbing direction; (c) 3D rendering of simulated director structures, (d) simulated POM textures with crossed polarizers along the rubbing direction; and (e) simulated POM textures with crossed polarizers at 45 °C with the rubbing direction. The lighter the color of the columns, the closer is the director to the rubbing direction. Dark green columns indicate director perpendicular to the rubbing.

On heating in the N_F_ phase at 28 °C (see Figure 7A), the texture is darker in between crossed polarizers along the rubbing direction (Figure 7A­(a)) than when it makes 45° with respect to the rubbing (Figure 7A­(b)). This shows the majority of the director aligns along the rubbing direction except at the periphery where the director is tangential to avoid space charge due to the depolarization field. Additionally, there is a noticeable waviness in the transmittance. The simulation shown in Figure 7A­(c) reveals that this is due to a α_0_ ≈ π intrinsic twist along *z* that is sufficient to rotate the director by half a turn between the base and the dome, so that the dome and base director orientations are antiparallel. This half-twist cannot be achieved uniformly across the entire droplet cross section since the spherical cap does not admit a single-domain helical solution and the system instead resolves the frustration by partitioning the bulk into domains of clockwise and anticlockwise twist that coexist and meet along internal boundaries. At the base boundary, this mixed-twist structure terminates in two +1/2 disclination defects positioned at the droplet edges parallel to the rubbing direction. The simulated POM textures shown in Figure 7A­(d,e) are shown for the rubbing parallel to and at 45° with respect to the polarizers, respectively. They not only capture the wavy modulation but also reproduce the number of interference fringes observed in Figure 7A­(a,b). Details of the material parameters and simulated textures with cross-sectional views of the dome and base plates are shown in Figure S1 of Supporting Information.

In the SmZ_A_ phase on heating from the N_F_ phase, we see almost uniform textures being dark when the rubbing direction is parallel to the crossed polarizers (see Figure 7B­(a)) and uniformly bright with concentric interference rings (see Figure 7B­(b)). This texture is reproduced by the simulation shown in Figure 7B­(c) and reflected correctly in the simulated POM images shown in Figure 7B­(d,e). The uniform texture is likely related to the antiferroelectric nature of the phase, thus allowing uniform alignment even at the periphery without the creation of the depolarization field. We note here that at higher heating rates sometimes zigzag-type domains and wrinkling of the interference fringes (see Figures 5 and 6) appear that are likely due to the buckling of the smectic layers related to the decrease of the planar layer spacing.^33–35^ Details of the physical parameters and simulated textures with cross-sectional views of the dome and base plates are shown in S2 of Supporting Information.

In the N phase at 52 °C on heating from the N_F_ and N_X_ phases, we see the formation defect walls running mainly perpendicular to the rubbing direction (see Figure 7C­(a,b)) similar to that observed in conventional N droplets with uniform planar alignment at the base^36^ where at the air interface the director typically aligns perpendicular to the dome surface.^37–42^ In some cases, the director can also be tilted^42–46^ resulting in an azimuthally degenerate conical alignment. Evidenced from the stepwise decrease of the birefringence upon transition to the N phase (see Figure 4c), in our case the defect wall is due to the conical director alignment at the air interface. The transition to the conical alignment at the dome leads to the formation of oppositely splayed domains that are separated by the defect wall. This splay deformation may also be coupled to twist provided the twist elastic constant becomes smaller than of the splay. In this case, the wall may split to a splay-twist and a twist-splay walls. Indeed, sometimes single, sometimes double walls appeared in our experiments. Details of the physical parameters and simulated textures with cross-sectional views of the dome and base plates are shown in Figure S3 of Supporting Information.

On cooling the droplet from 52 °C (as shown in Figure 7C) to the SmZ_A_ phase, the walls are separated further from each other as shown in Figure 8A. This is likely due to the different layer alignment outside and in-between the walls. Outside the walls there is only director splay that is compatible with the bookshelf structure of smectic layers that appears to be more stable near the N phase. On the other hand, in-between the walls the twisted director structure is not compatible with the bookshelf layers as that would cause twist-grain boundary phase. Instead, the layers adopt planar alignment. Accordingly, the two walls separate bookshelf-planar and planar-bookshelf structures. As the planar layer structure is getting more and more stable toward lower temperature, the thickness of the range separating the walls increases on cooling as it can be seen in Figure 8A. Our simulation agrees with this scenario (see Figure 8A­(c,d,e)). Details of the simulations with material parameters, initial conditions, and 2D cross sections of the top dome and bottom base plate are described in Figure S4 of Supporting Information.

Cooling further to the N_F_ phase, the SmZ_A_ layers disappear and only regions separating twisted and nontwisted domains remain, as shown in Figure 8B. Details of the simulations with material parameters, initial conditions, and 2D cross sections of the top dome and bottom base plate are described in Figure S5 of Supporting Information.

## 4 Conclusions

In this study, we have shown that droplets of a room-temperature ferroelectric nematogen mixture FNLC 919 printed on glass substrates with a uniformly rubbed planar alignment layer lead to bifocal lens arrays. The focal lengths of the lenses depend on the polarization direction of the light, corresponding to $f_{o}=\frac{R}{n_{o}-1}$ and $f_{e}=\frac{R}{n_{e}-1}$ , where *n*_o_(*n*_e_) is the ordinary (extraordinary) refractive index. These equations correspond to the Lensmaker’s equation for a plano-convex lens with uniform anisotropic medium. It is a good approximation when the director profile varies in a length scale much larger than that of the wavelength of the visible light. This is true everywhere in our lenses outside defects. To observe both *f*_o_ and *f*_e_ require planar alignment conditions in the N_F_ and SmZ_A_ phases on both the base plane and on the dome of the droplets. Our experimental studies corroborated by finite element simulations show that in these two phases the shape of the droplets is spherical cap and the textures do not contain defects as long as the sample is not heated to the nematic phase (in the studied material up to 45 °C). The observed bifocal behavior of these printable sessile droplets may have applications in autostereoscopy that can add 3D perception without the use of special headgear and glasses. Additionally, such lens arrays can improve depth of focus (DOF) without the need of repeated photolithography with multiple photomasks and alignment to define microposts of different thicknesses.

Future work will focus on the stabilization and electrical switching of bifocal microlens arrays. One avenue is to study ferroelectric nematic mesogens that are printed at elevated temperatures but vitrified at room temperature. Another direction of work is polymer stabilization of the lens array, which simultaneously enables electrical switching of the director structure and consequently of the focal length.

## Supplementary Material



## Data Availability

The data that support the findings of this study are available from the corresponding author upon reasonable request. The main data supporting the results of this study are available within the article and its Supporting Information Additional raw data and simulation files are available on GitHub.^47^

## Notes

Free Energy terms, Initial Conditions, and Materials Parameters considered in the simulations; director structures in the N_F_, SmZ_A,_ and N phases obtained by the simulations; description of the Polarized Optical Microscopy Image of the Simulated Director Field; and references used in the Supporting Information (PDF)
