# Supplementary material for: Printable Bifocal Microlenses from Ferroelectric Nematic Liquid Crystal Droplets
Source: ACS Appl Mater Interfaces. 2026 Jun 8;18(23):33426–36. doi: 10.1021/acsami.6c06633 (PMC13288378; doi:10.1021/acsami.6c06633)
Supplement: Supplementary file 1 [file am6c06633_si_001.pdf]

Supporting Information

Printable Bifocal Microlenses from Ferroelectric Nematic Liquid Crystal Droplets

Manisha Talwar[1], Zakaria Siddiquee[1], Antal Jákli[1,2,]*

[1]Department of Physics, Kent State University, Kent OH, 44242, USA

[2]Advanced Materials and Liquid Crystal Institute, Kent State University, Kent OH, 44242, USA

[*]Author for correspondence: ajakli@kent.edu

I. Free Energy Terms

When a liquid crystal droplet is deposited on a flat substrate, the collective orientation of the constituent molecules described by the "director" $\hat{n}$ field adopts a configuration governed by the competing influences of elastic distortions, surface anchoring and electric polarization. Predicting this equilibrium configuration, and the dynamic pathway by which the system reaches it, is the central aim of the employed finite element implemented in DOLFINx 0.9.0 [1–4] with PETSc [5,6] providing the linear and nonlinear algebra backend.

The liquid crystal will evolve toward the configuration that minimizes its total free energy, analogous to a mechanical system settling into its lowest potential energy state. The scalar free energy function $F = \int f \, dV$ is the volume integral of the total free energy density f that quantifies the elastic distortions, surface anchoring, and, where applicable, electric or smectic interactions. The simulation drives the system toward the minimum of the free energy by evolving the director field along the steepest descent of this energy landscape. This approach is known as gradient-flow and is governed by the Ginzburg-Landau solver:

$$\gamma \cdot \frac{d\hat{n}}{dt} = -\frac{\delta F}{\delta \hat{n}} \tag{S1}$$

Here $\hat{n}$ is the director field, which encodes both the orientation at every point in the droplet; γ is a viscosity-like coefficient that sets the rate of relaxation; and $\delta F / \delta \hat{n}$ is the variational derivative which is the measure of how sensitively the total energy responds to a local perturbation in $\hat{n}$. The negative sign ensures the field evolves in the direction of steepest energy descent. Because the droplet is three-dimensional and $\hat{n}$ varies continuously through space, this equation takes the form of a partial differential equation (PDE). Solving it numerically requires discretisation in both space and time.

Within each element, $\hat{n}$ is represented by piecewise-linear basis functions. This is the finite element method (FEM), implemented here via the FEniCSx library. The continuous PDE is thereby converted into a large but finite system of algebraic equations, with one set of unknowns per mesh node.

In time, the simulation advances in discrete 10^4 steps of size $dt = 10^{-3}s$ for a total maximum of $10^4 s$. The time derivative $d\hat{n}/dt$ is approximated using a Backward Differentiation Formula (BDF). The first-time step employs BDF1 (Backward Euler) [7], a first-order scheme chosen for its robustness when starting from an arbitrary initial condition. All subsequent steps use BDF2, a second-order scheme that draws on the two most recent solutions to achieve greater accuracy at comparable computational cost.

At each time step, the discretized system is nonlinear and cannot be solved by direct matrix inversion. Newton's method is therefore employed: beginning from the previous solution as an initial estimate, the solver iteratively refines $\hat{n}$ until the residual value (the degree to which the governing equation is not yet satisfied) falls below a prescribed tolerance. Each Newton iteration reduces to a single linear system, which is solved by direct Lower-Upper (LU) factorization using the MUMPS library.[8,9] This inner-outer structure, with Newton iterations driving convergence and direct solves handling the linear sub-steps, is managed by PETSc's framework.

Given the breadth of liquid crystal phase behavior, capturing all relevant physics simultaneously in one computational model is a notoriously difficult task. The problem is therefore decomposed into three simulation packages, each corresponding to a distinct material phase: a standard nematic, an antiferroelectric smectic, and a ferroelectric nematic.[10] Each package initializes the director field from a prescribed starting configuration and advances it forward in time until the system reaches mechanical equilibrium. All three share a common mesh geometry, time-stepping scheme, and solver architecture, differing only in the physical model.

All three packages share a bulk free energy density f that controls how strongly the director (molecular direction averaged in a microscopic volume containing large enough molecules to be considered continuum but small enough to fit in one grid) is ordered.

In the uniaxial non-polar N phase, the elastic contributions only contain the splay (K_1), twist (K_2) and bend (K_3) Frank director distortion terms, so that the free energy density is[11,12]

$$f_d = \frac{1}{2}K_1(\nabla\cdot\hat{n})^2 + \frac{1}{2}K_2\big(\hat{n}\cdot(\nabla\times\hat{n})\big)^2 + \frac{1}{2}K_3\big(\hat{n}\times(\nabla\times\hat{n})\big)^2 \qquad (S2)$$

In the ferroelectric N_F phase, the director is polar ($\vec{n} = \frac{\vec{P}}{|\vec{P}|}$) and one has to add another term to ($S2$) that is a linear function of the deformation[12], $f_F = -\frac{1}{2}K_F\vec{\nabla}\cdot\vec{n}$. This term expresses that the presence of ferroelectric polarization leads to a spontaneous splay is analogous to a splay flexoelectric term $f_{sf} = -\gamma_s(\vec{n}\cdot\vec{P})(\vec{\nabla}\cdot\vec{n})$ [13] where γ_s is related to the splay flexoelectric coupling constant e_1 as $e_1 = \gamma_s\varepsilon_o\varepsilon$. [14] As K_F has a unit of N/m such as the surface tension ($\sigma\sim10^{-2}N/m$) in liquid crystals) and surface anchoring ($10^{-3}\,N/m \le W \le 10^{-6}N/m$) coefficient. Since the shape of our ferroelectric nematic sessile droplets in good approximation have spherical cap shape, we expect that $K_F \ll 10^{-3}N/m$. From light-induced thermomechanical effects, it was estimated that $\frac{\partial K_F}{\partial T}\sim10^{-4}N/(m\cdot K)$.[15] From the temperature dependences of the ferroelectric polarization, we assuming that K_F reaches maximum in a few degrees below the transition to the N_F phase, thus $K_F\sim10^{-4}N/m$. Assuming $\varepsilon\sim10^2$, the magnitudes of f_F and f_{sf} are comparable for $\gamma_s\sim2\cdot10^{-3}V$, i.e., when $e_1\sim2\cdot10^{-11}C/m$. These two f_F and f_{sf} energy density terms can be lumped to an effective term,

$$f_{\tilde{F}} = -\frac{1}{2}\widetilde{K}_F\vec{\nabla}\cdot\vec{n} \tag{S3}$$

Here $\widetilde{K}_F = K_F + \frac{e_1}{\varepsilon_o\varepsilon}(\vec{n}\cdot\vec{P})$ is the effective polar Frank elastic energy term. We note that Eq.(S3) can also be included in Eq.(S2) by modifying the splay elastic term as $\frac{1}{2}K_1(\nabla\cdot\vec{n} - S_o)^2$, where $S_o = \frac{\widetilde{K}_F}{K_1}$ is the curvature of spontaneous splay.

In *the SmZ_A phase*, due to the layered structure, there is an additional free energy density term f_l. This considers that a compression or dilation of the layers by a distance u along the layer normal direction $\hat{k}$ is penalized by a free energy density

$$f_l = \frac{1}{2}\cdot B\cdot\left(\hat{k}\cdot\vec{\nabla}u\right)^2, \tag{S4}$$

where B is the layer compression modulus. We choose $\hat{k}$ direction appropriately to reflect experimental observations for DIO[16], where on cooling into the smectic phase from the N phase yields a preferential bookshelf structure with the layers parallel to the base plate along the rubbing direction, and on heating from the N_F phase, a planar alignment of layers being perpendicular to the base plate appears.[17] In conventional orthogonal smectic A liquid crystals, the layer compression modulus is in the order of 1 MPa [18], whereas in the SmZ_A phase it is estimated from dynamic light scattering measurements to be $B \approx 40\ kPa$.[19]

Concerning the boundary conditions, we have the following considerations.

(i) At the base, there is a preferred azimuthal direction along the rubbing direction $\hat{R}$, penalizing the departure from the rubbing direction by an energy per unit area term,

$$G_{ba} = -\frac{1}{2}\,W_{ba}\left(\hat{n}\cdot\hat{R}\right)^2, \tag{S5}$$

where W_{ba} is the azimuthal anchoring strength on the base.[20] Additionally, there is a zenithal anchoring term with anchoring strength W_{bz}that penalizes a departure from a planar alignment whereby the director prefers to be perpendicular to the base normal $\vec{k}_b$, i.e.,

$$G_{bp} = \frac{1}{2}\,W_{bz}\left(\hat{n}\cdot\vec{k}_b\right)^{\mathbf{2}} \tag{S6}$$

(ii) At the air-LC interface (dome boundary), all azimuthal directions are equivalent thus we only deal with the zenithal anchoring energy per unit area

$$G_d = \pm\frac{1}{2}\,W_d\left(\hat{n}\cdot\vec{k}_d\right)^{\mathbf{2}}, \tag{S7}$$

where $\vec{k}_d$ is the outward-pointing normal vector of the curved air-LC interface (dome) and W_d is the anchoring strength in N/m^2 unit. For positive sign, this energy is minimized when the director is parallel to the droplet's curved surface (planar alignment), while in case of negative sign, this energy is minimized when the director is perpendicular to the droplet's curved surface (homeotropic alignment).

At the contact line, where the dome and base surfaces meet, the mesh resolution is locally increased and the surface normal transitions smoothly to become parallel to the substrate. This geometric treatment ensures the dome anchoring condition naturally enforces tangential alignment at the contact line without requiring a separate boundary condition to be imposed there explicitly.

We also note that the N_F phase with degenerate boundary conditions exhibits a spatially varying intrinsic (spontaneous) twist driven by polar molecular interactions rather than chirality.[21,22] This is modeled by introducing a preferred twist wavenumber q_P that depends on the local dome height $h(r)$:

$$f_{it} = \frac{1}{2} \cdot K_2 \cdot (\vec{n} \cdot (\nabla \times \vec{n}) - q_P)^2 \tag{S8}$$

where $q_P = \alpha_0 / h(r)$, and α_0 is the number of full twists turns across the droplet height. When $\alpha_0 = 0$, the standard Oseen-Frank twist term in Eq. (S2) is recovered.

The equilibrium director configuration can be determined by the minimum condition of the total free energy that is the sum of the volume integrals of Eqs. (S2-S4) and surface integrals of Eqs. (S5-S11).

II. Initial Conditions

The choice of initial condition has a significant consequence for gradient-descent simulations. Two systems starting from different initial configurations will, in general, converge to different local energy minima - both in simulation and in experiment. Since the present simulations do not model the phase transition dynamics explicitly, the initial conditions are instead chosen to mimic the physical state from which each phase naturally emerges, either upon heating or upon cooling.

Upon heating, the phase sequence runs from crystal through N_F and SmZ_A to N. The initial conditions therefore follow this ordering:

- N_F is initialized from a crystal state.
- SmZ_A is initialized from the converged final state of the N_F (heating) simulation.
- N is initialized from the converged final state of the SmZ_A (heating) simulation.

Upon cooling, the sequence is reversed, beginning from the isotropic phase:

- N is initialized from an isotropic state.
- SmZ_A is initialized from the converged final state of the N (cooling) simulation with two extra Bloch walls added manually.
- N_F is initialized from the converged final state of the SmZ_A (cooling) simulation with random director polarization $\vec{P}$.

The crystal initial condition sets $\hat{n}$ uniformly along the substrate rubbing direction at the base. Through the droplet bulk and at the dome surface, the field is projected onto the local surface tangent plane so that it follows the spherical curvature while remaining aligned as closely as possible with the rubbing direction.

The isotropic initial condition is available in two variants. In the first, $\hat{n}$ is fully randomised throughout the entire droplet. In the second, $\hat{n}$ is randomised at the base but oriented radially

outward, parallel to the local surface normal, in the bulk and at the dome. The motivation for this second variant is physical: near the nematic-to-isotropic transition, the air–LC interface is observed experimentally to favor homeotropic (normal) anchoring. Upon cooling through this transition, this homeotropic dome configuration also seeds a disclination line through the center of the droplet, a topological feature that would not emerge from a fully randomized starting state. The base anchoring strength is set to $W_{ba} = W_{bz} = W_b = 5 \times 10^{-5}\, J/m^2$ and the dome anchoring strength is five times weaker $W_d = 1 \times 10^{-5}\, J/m^2$, reflecting the stronger influence of the rubbed substrate relative to the free air–LC interface.

III. Material Parameters

In the *N_F phase* at T=28°C, from recent estimate based on electrochomism of chiral room temperature FNLC material[23] we chose the splay elastic constant to be $K_1 = 400\, pN$ and, by extrapolating from elastic constants measured at the SmZ_A-N_F transition temperature by Paul et al[24] , we estimate the Frank elastic constants were chosen to be $K_2 = 10\, pN$ and $K_3 = 13\, pN$ and the rotational viscosity $\gamma = 10\, Pa \cdot s$. Additionally, based on our results shown in Figure 3(a) of the main document, the contact angle was taken as $\theta = 18°$.

In the SmZ_A phase at T=36°C, based on elastic constant measurement results by Paul et al[24] the Frank elastic constants were chosen to be $K_1 = 10\, pN$; $K_2 = 2\, pN$ and $K_3 = 3\, pN$ and the rotational viscosity $\gamma = 0.36\, Pa \cdot s$ was used. Additionally, based on our results shown in Figure 3(a) of the main document, the contact angle was taken as $\theta = 18°$.

In the *N phase* at T $= 52$°C, based on elastic constant measurement results by Paul et al[24] the Frank elastic constants were chosen to be $K_1 = 5\, pN$; $K_2 = 1.5\, pN$ and $K_3 = 3\, pN$ and the rotational viscosity $\gamma = 0.03\, Pa \cdot s$ was used. Additionally, based on our results shown in Figure 3(a) of the main document, the contact angle was taken as $\theta = 15°$.

IV. Simulated cross sectional director structures

With these for a droplet of radius $a = 127\ \mu m$ and assuming $\theta_{\mathrm{pretilt}} = 0°$ the simulation results of the director structures at 28°C, 36°C and 52°C on heating and on cooling at 36°C and 28°C are shown in Figure 7 and Figure 8 of the main document, respectively. The cross-sectional director

structures at the base and on the dome are shown in Figure S1 at 28 °C in the N_F phase with a spontaneous twist of $\alpha_0 = \pi$.

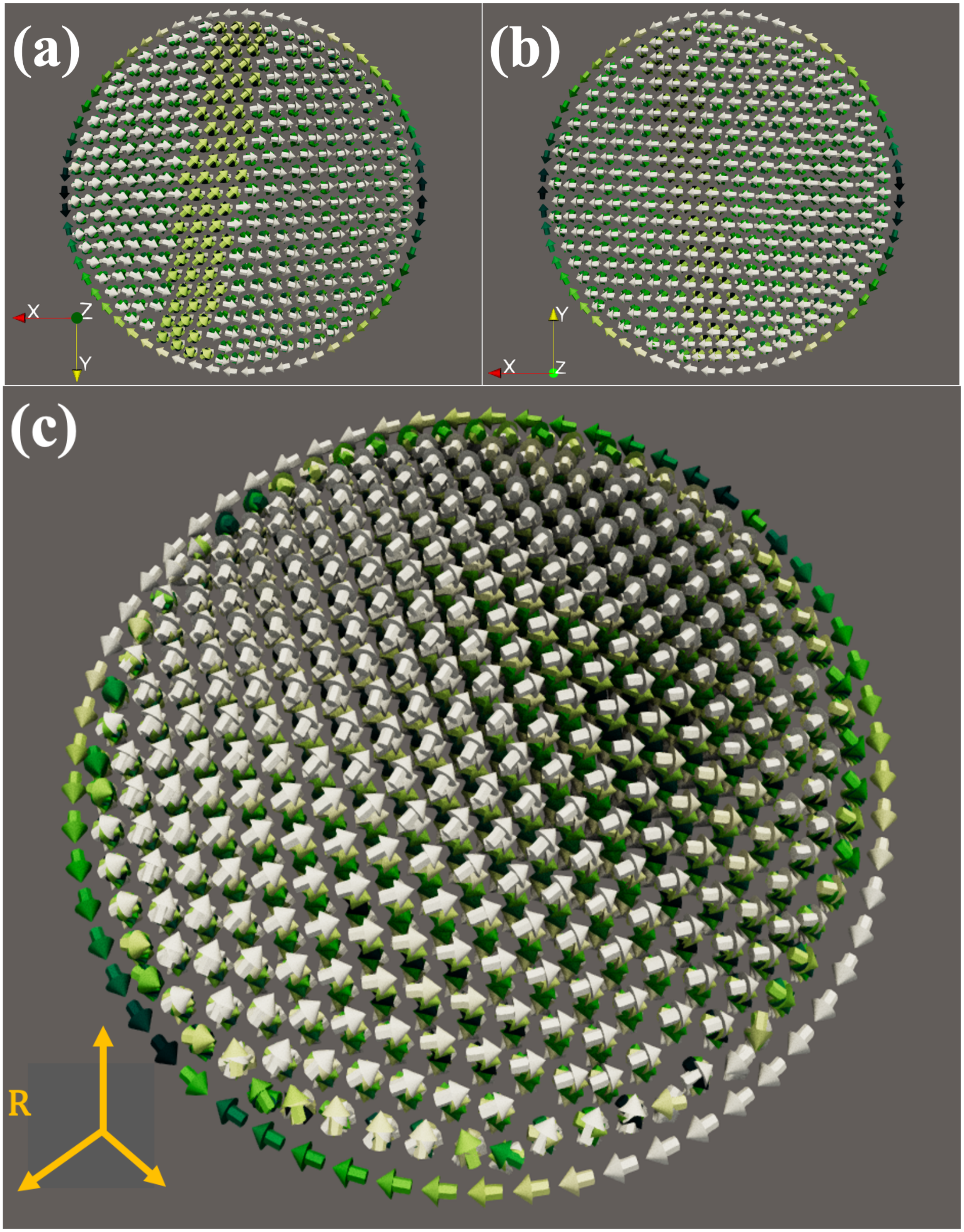

***Figure S1**: Director field configuration for N_F phase simulation with intrinsic twist $\alpha_0 = \pi$. (a) Cross-section of the dome; (b) Cross-section of the base plane. (c) 3D view (see also Figure 7A(c)). Rubbing direction is along x-axis.*

Figure S2 shows the cross-section director field at 36 °C in the SmZ_A phase using the N_F structure as initial condition. To replicate the antiferroelectric smectic configuration with the layer normal being vertical, i.e., the layer planes lie horizontally within the droplet. The equilibrium configuration, shown in, preserves the smooth, nematic-like director field inherited from the N_F phase.

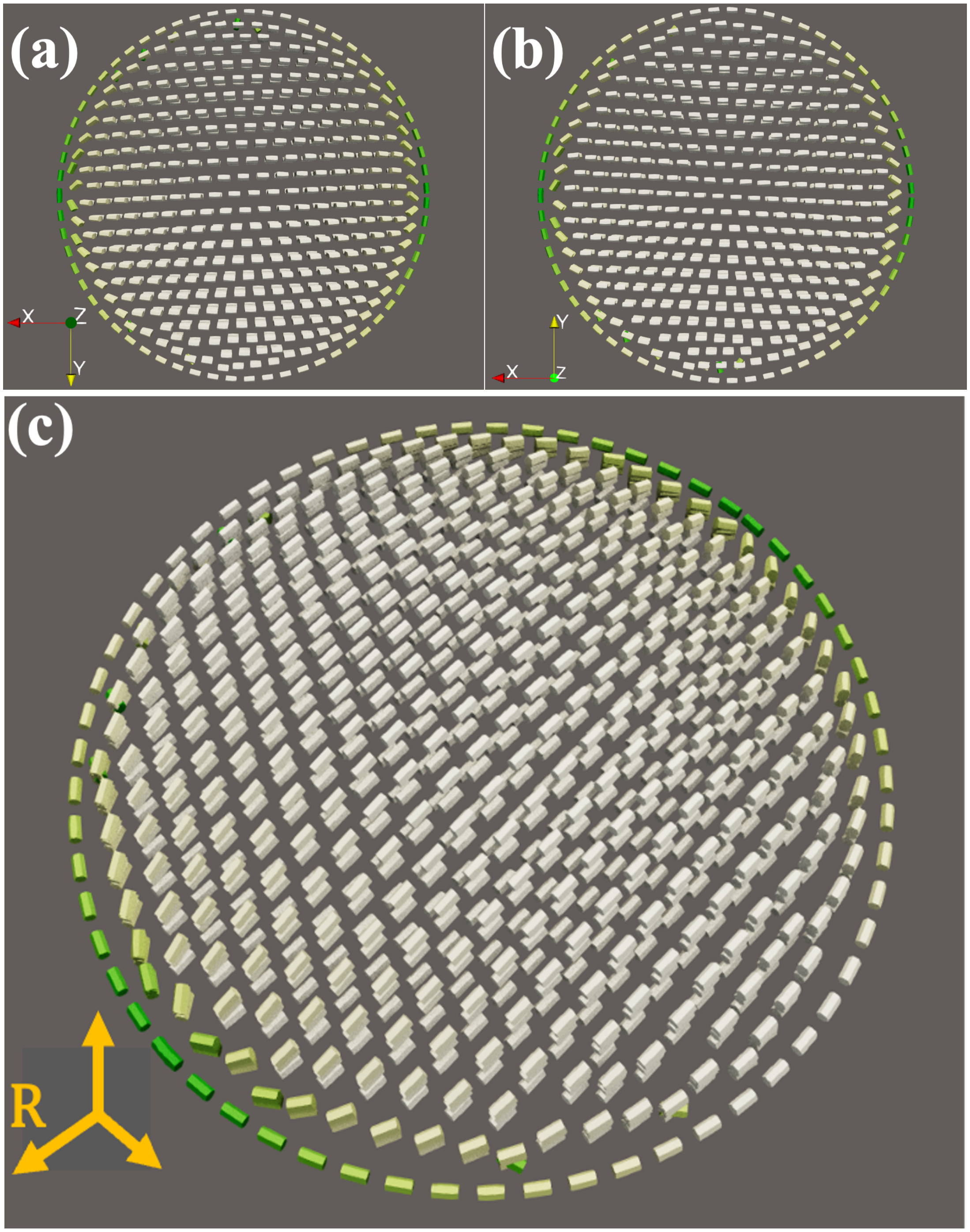

***Figure S2**: Director field configuration for SmZ_A phase simulation (heating) with ferroelectric nematic phase initial conditions. (a) Cross-section of the dome; (b) Cross-section of the base plane.(c) 3D view (see also Figure 7B(c)). Rubbing direction is along x-axis*

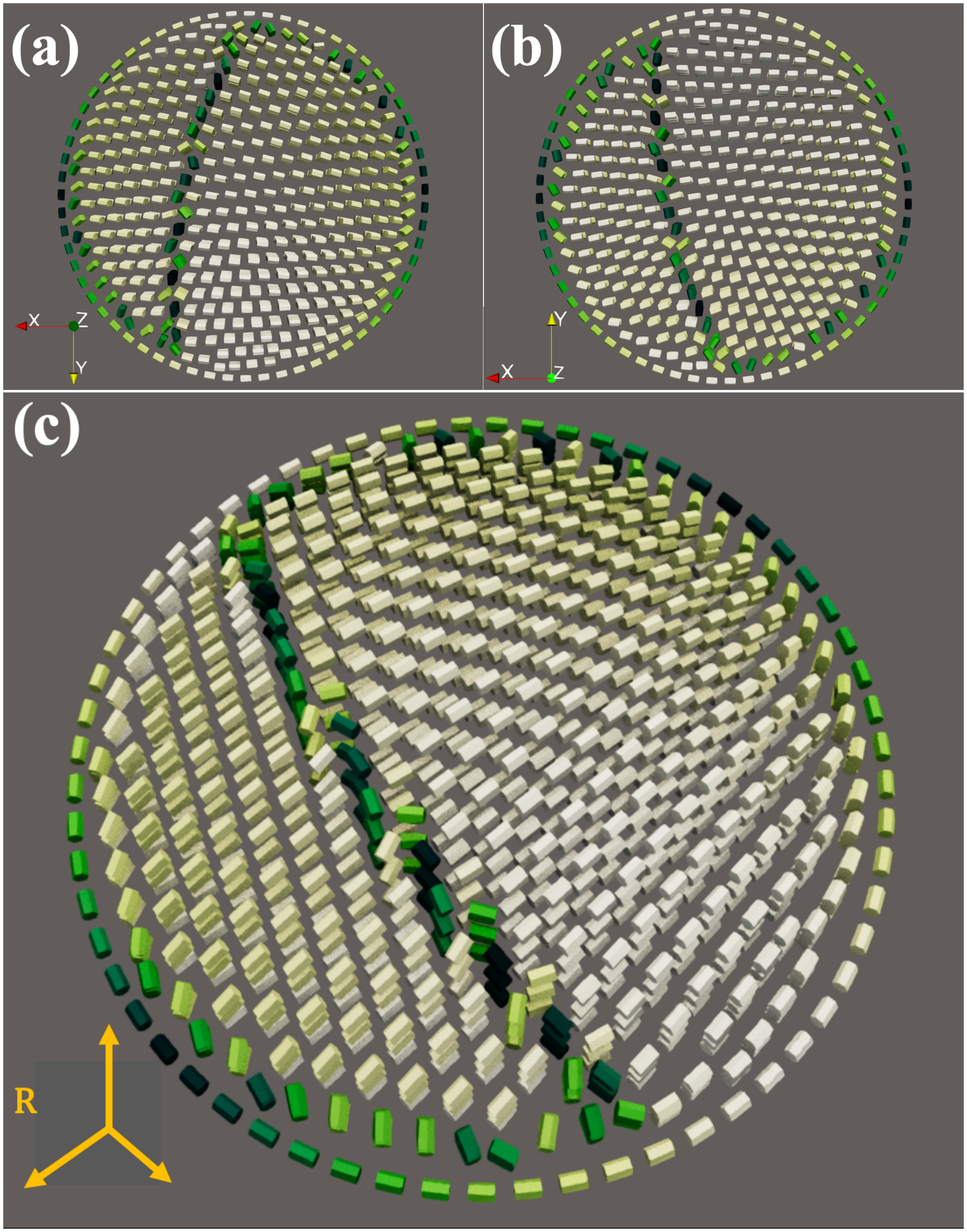

Figure S3: *Director field configuration for nematic phase simulation (heating) with* SmZ_A *phase initial conditions. (a) Cross-section of the dome; (b) Cross-section of the base plane. (c) 3D view (see also Figure 7C(c)). Rubbing direction is along x-axis.*

Cross sections of the director field at 52°C in the N phase with an initial condition corresponding to the uniform director field of the SmZ_A phase is shown in Figure S3. After long time these lines

move toward one of the edges to decrease the energy of the wall and they disappear after very long time one sees a defect wall forming in the center of the droplet essentially perpendicular to the rubbing direction.

The cross-sections on cooling from 52 °C are shown in **Figure S4** at 36 °C in the SmZ_A phase with initial condition corresponding to the texture in Figure S3. Outside the defect walls the director follows the rubbing direction at the base and curves toward the dome, whereas in between the walls the director is rotated.

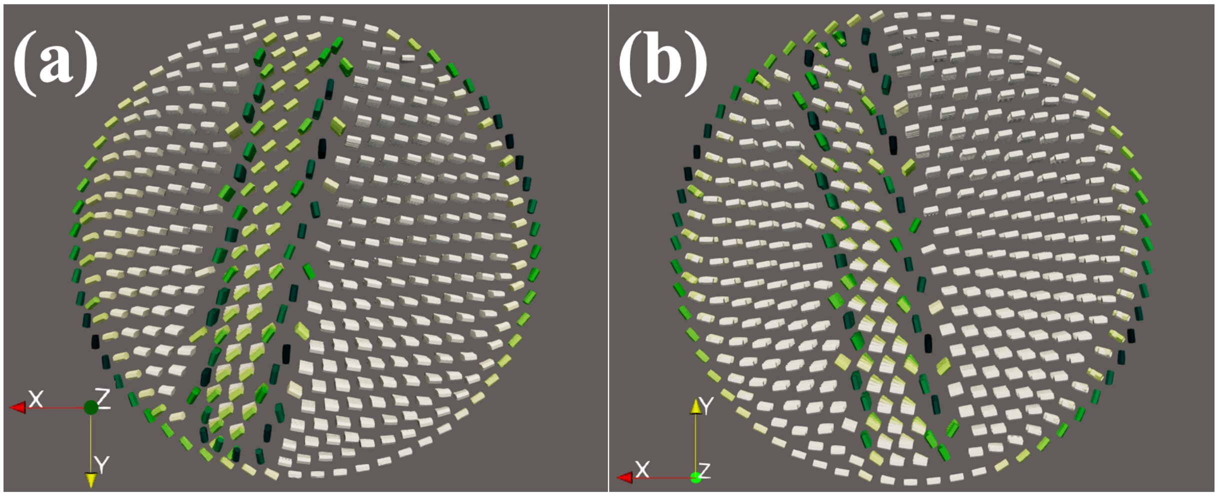

Figure S4: *Director field configuration for SmZ_A phase simulation (cooling) with nematic phase initial conditions. (a) Cross-section of the dome; (b) Cross-section of the base plane. Rubbing direction is along x-axis. For 3D view see Figure 7A(c).*

The cross-sections at 28°C in the N_F phase with initial condition corresponding to the texture shown in Figure S4 are shown in **Figure S5**. The resulting texture resembles that shown in Figure S4 except that the defect walls separating the twisted and non-twisted areas disappear.

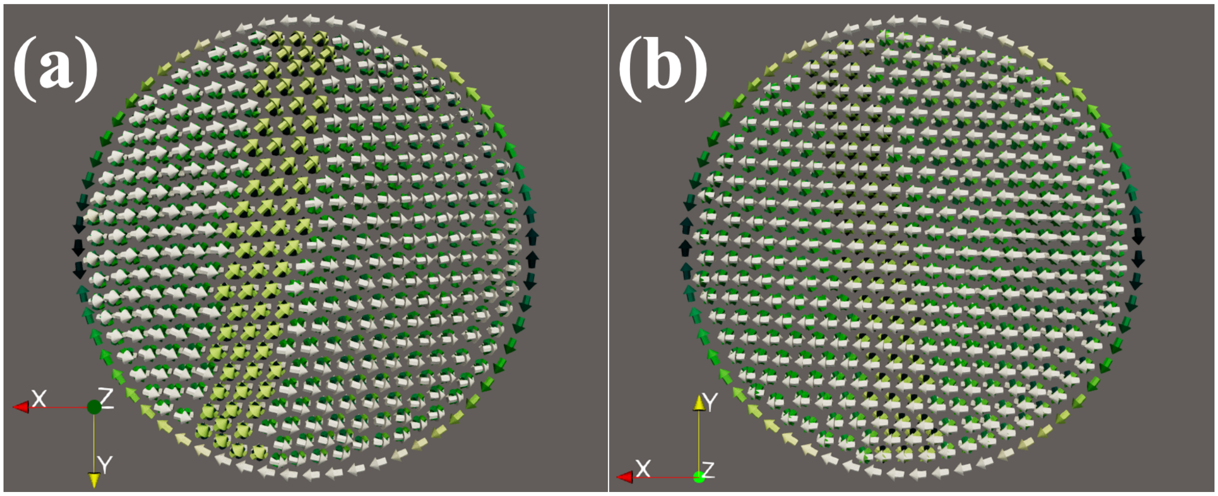

Figure S5: *Director field configuration for N_F phase simulation with SmZ_A phase initial conditions (cooling). (a) Cross-section of the dome; (b) Cross-section of the base plane. Rubbing direction is along x-axis. For 3D view see Figure 7B(c).*

V. Polarized Optical Microscopy Image of the Simulated Director Field

Polarized optical microscopy (POM) is the primary experimental technique by which the internal structure of a liquid crystal droplet is made visible. When the sample is placed between two crossed polarizing filters, namely the polarizer and the analyzer oriented at 90° to one another, regions where the director lies parallel or perpendicular to the polarizer appear dark, while regions where the director takes an intermediate orientation transmit light and appear colored. The colors arise from optical birefringence: a uniaxial liquid crystal possesses two distinct refractive indices, n_e for light polarized along the short-axis and n_o for light polarized perpendicular to it. As a wave traverses the sample, the two orthogonal polarization components accumulate different optical path lengths and emerge with a phase difference, the retardation, that determines which wavelengths of white light interfere constructively and which destructively at the analyzer. Because the retardation depends on both the local director orientation and the local sample thickness, the resulting color pattern encodes the three-dimensional director field in a single two-dimensional image. We used Jones Matrix method and JM_POM package to simulate the POM image of the simulated director structure of the droplets. This process from the computed director fields, reproducing the images that would be observed experimentally without requiring any physical sample.

<u>*IV.1. The Jones Calculus*</u>

To simulate light propagation through the birefringent droplet, the Jones calculus formalism is employed. In Jones calculus, the electric field of a monochromatic plane wave propagating along the z axis, which is the optical axis of the microscope, is represented as a two-component complex column vector known as the Jones vector:

$$E = \begin{bmatrix} E_x \\ E_y \end{bmatrix} = \begin{bmatrix} |E_x| e^{i\varphi x} \\ |E_y| e^{i\varphi x} \end{bmatrix} \qquad (S9)$$

Here E_x and E_y encode the amplitude and relative phase of the x and y polarized field components, fully characterizing the polarization state. Linear polarization at angle θ to the x axis corresponds to the real vector $[cos\ \theta, sin\ \theta]$; a horizontal polarizer transmits only the x component and is represented by the projector $[1, 0]$. Any optical element that acts linearly on polarization, such as a polarizer, a wave retarder, or a birefringent crystal slab, is represented by a 2×2 complex matrix, the *Jones matrix*, and the combined action of a stack of optical elements is obtained by successive left multiplication of the Jones vector.

A liquid crystal layer of thickness d, with its optic axis tilted at polar angle θ from the z axis and oriented at azimuthal angle ϕ in the xy plane, behaves as a phase retarder. The effective extraordinary refractive index seen by the wave depends on how steeply the director is tilted relative to the propagation direction:

$$n_{eff} = \frac{n_e \cdot n_o}{\sqrt{n_e^2 cos^2\theta + n_o^2 sin^2\theta}} \qquad (S10)$$

When the director lies fully in plane ($\theta = 90°$), n_{eff} reaches its maximum value n_e and the layer is maximally birefringent. When the director is homeotropic, pointing straight up along the optic axis at $\theta = 0°$, n_{eff} collapses to n_o, the birefringence vanishes, and the layer is effectively

isotropic regardless of the azimuthal angle. The phase retardation accumulated across the layer at wavelength λ is:

$$\delta = \frac{2\pi \left(n_{eff} - n_o\right)d}{\lambda} \qquad (S11)$$

and the corresponding Jones matrix for the layer, referred to the laboratory frame, is constructed by rotating the field into the fast axis frame of the retarder, applying a diagonal phase advance of $exp(-i\delta/2)$ on the fast (extraordinary) axis and $exp(+i\delta/2)$ on the slow (ordinary) axis, then rotating back:

$$J(\theta, \delta) = R(-\theta) \cdot Retarder(\delta) \cdot R(\theta) \qquad (S12)$$

where $R(\theta) = \begin{bmatrix} cos\,\theta, & sin\,\theta \\ -sin\,\theta, & cos\,\theta \end{bmatrix}$ is the standard rotation matrix. For a sample divided into N layers along the propagation direction, the total Jones matrix is the ordered product $J_{total} = J_N \times \ldots \times J_2 \times J_1$, with layer 1 encountered first by the incoming light. The transmitted intensity after the crossed analyser is then:

$$I(\lambda) = \left|E_{analyzer} \cdot J_{total}(\lambda) \cdot E_{polariser}\right|^2 \qquad (S13)$$

where $E_{polariser} = [cos\,\psi, sin\,\psi]$ is the Jones vector for light through the polariser at angle ψ, and $E_{analyzer}$ projects onto the analyzer direction at $\psi + 90°$. This is computed separately for each wavelength sampled across the visible spectrum, and the results are averaged to obtain the broadband intensity at each pixel.

In the JM_POM package this calculation is carried out in native machine code using Numba's JIT decorator for performance. The function propagates the Jones vector layer by layer in an explicit loop, accumulating the rotation and retardation operations in place on the four real components of the complex field vector, rather than constructing full 2×2 matrices, a form that avoids unnecessary memory allocation and is well suited to Numba's scalar optimisation. The spectrum is sampled at twenty wavelengths between 380 and 750 nm, and the per wavelength intensities are summed and normalised to yield the final scalar intensity for each pixel.

One practical complication arises from the finite resolution of the FEM mesh near the substrate. The coarse mesh does not resolve the anchoring boundary layer at the base of the droplet, leaving a thin region at the bottom of each vertical column without reliable director data. To compensate, a set of dummy layers is prepended to each column before the mesh data begins, each assigned a uniform director pointing along the x axis, consistent with the rubbing direction of the substrate. The phase accumulated in these dummy layers is included in the Jones product, so the simulated image reflects the full optical path through the droplet including the otherwise unresolved anchoring region near the glass.

A further subtlety concerns the sign of the director extracted from each layer. The polarization field P returned by the FEM solver carries a definite sign at each mesh node, but the optical calculation depends only on the headless director $\hat{n}$, and in the nematic case, extracting $\hat{n}$ from a Q-tensor by eigenvector decomposition yields an arbitrary sign per layer. If adjacent layers happen to point in opposite hemispheres, the computed twist between them is spurious rather than physical. The

pipeline corrects this by walking down each pixel column after interpolation and flipping the sign of any layer whose director has a negative dot product with the layer immediately below it, thereby enforcing a consistent orientation convention throughout the column before the Jones product is computed.

IV.2. The Michel-Lévy Interference Color Chart

The Jones calculus described above returns a scalar intensity for each pixel at each polarizer angle; this scalar conveys contrast but carries no color information. Color enters through the interference of white light across the visible spectrum, and the relationship between retardation and the color an observer perceives is codified in the Michel-Lévy chart.

For a uniform birefringent slab of total optical path difference (OPD) $\Gamma = \Delta n \times d$, the transmitted intensity under crossed polarisers with the optic axis at 45° is $sin^2(\pi\Gamma/\lambda)$ for monochromatic light at wavelength λ. Under polychromatic white illumination, each wavelength acquires its own independent sin^2 weighting. At very small retardations, all wavelengths are equally suppressed, and the field appears dark grey. As Γ increases through a few hundred nanometres, the suppression pattern shifts across the spectrum: wavelengths near the centre of the visible range are extinguished while others pass, producing the pale yellows and whites of the first order. Near $\Gamma \approx 550\, nm$, green is suppressed and the field takes on the vivid red violet of the transition from first to second order, the most recognizable color on the chart and commonly used as a landmark by experimentalists. Higher orders continue the sequence through blue, green, yellow, and pink, but the colors become progressively paler and more washed out as many wavelengths interfere simultaneously and their contributions partially cancel.

Within the simulation, every pixel (i,j) is associated with an OPD value determined by the column-averaged effective birefringence and the local droplet height $h(i,j)$:

$$\Gamma(i,j) = \int_0^{h(i,j)} \Delta n_{eff}^{(i,j)}(z) \cdot dz\,(i,j) \qquad (S14)$$

where$\Delta n_{eff}^{(i,j)}(z) = n_{eff}^{(i,j)} - n_o$ is the effective birefringence of pixel (i,j) at vertical position z, and $h(i,j)$ is obtained from the spherical-cap height profile. The full array of retardation values is passed as a single batch which evaluates the Michel-Lévy color at every pixel simultaneously, producing the base color map. This base color map reflects only the local retardation; it is independent of the polarizer angle and is computed once before any Jones calculus is run. When a POM frame is assembled at a given polarizer orientation, the Jones intensity for that angle modulates this color pixel by pixel: $final\ image\ (i,j) = base\ color\ image(i,j) \times intensity\ map(i,j)$, so that the color encodes the local birefringence while the contrast, consisting of the dark extinction brushes and crosses characteristic of each director configuration, arises from the Jones matrix calculation. This factorization mirrors what happens physically in the microscope: the color is set by the material's birefringence and thickness, while the intensity is set by the relative orientation of the director with respect to the polarizers.

IV.3. Commission Internationale de l'Éclairage (CIE) Color Matching

Converting a physical spectrum, specifically a white light source modulated by $sin^2(\pi\Gamma/\lambda)$ across the visible range, into an RGB value that a computer display can render requires accounting for how the human visual system integrates spectral information. The human eye contains three classes

of cone photoreceptor with overlapping spectral sensitivities, and it is the ratio of their responses, not the detailed shape of the spectrum, that determines perceived color. Two physically different spectra that produce the same cone response triplet are perceived as identical in color, a phenomenon known as metamerism.

The CIE 1931 standard observer formalizes this by defining three color matching functions, conventionally written $\bar{x}(\lambda), \bar{y}(\lambda), and\ \bar{z}(\lambda)$, which are empirically derived sensitivity curves that represent the integrated response of an average observer's visual system. Given a spectral power distribution $S(\lambda)$, the perceived color is reduced to three tristimulus values by integration:

$$X = \int S(\lambda)\,\bar{x}(\lambda)\,d\lambda$$
$$Y = \int S(\lambda)\bar{y}(\lambda)d\lambda \qquad (S15)$$
$$Z = \int S(\lambda)\,\bar{z}(\lambda)\,d\lambda$$

For the Michel-Lévy color chart, the effective spectrum at optical part difference Γ is $S(\lambda;\ \Gamma) = sin^2(\pi\Gamma/\lambda)$, assuming a spectrally flat illumination source. The tristimulus values computed from this spectrum are then mapped to display ready sRGB values through the standard linear XYZ to sRGB matrix [25]:

$$\begin{bmatrix} R \\ G \\ B \end{bmatrix} = \begin{bmatrix} 3.2406 & -1.5372 & -0.4986 \\ -0.9689 & 1.8758 & 0.0415 \\ 0.0557 & -0.2040 & 1.0570 \end{bmatrix} \begin{bmatrix} X \\ Y \\ Z \end{bmatrix} \qquad (S16)$$

followed by gamma correction at exponent $1/\gamma$, with $\gamma = 2.2$ for the sRGB standard, to convert from linear light intensities to the nonlinear values that displays expect.

The color matching functions $\bar{x}, \bar{y}, \bar{z}$ have no simple analytical form, as they were determined through careful psychophysical experiments and are available only as tabulated data. In particular, $\bar{x}(\lambda)$ has a secondary sensitivity lobe in the violet near $450\ nm$ that no smooth polynomial can capture faithfully, and errors there would distort the first order violet region of the chart, which is precisely where the transition between crystal and nematic phases is most clearly distinguished. The simulation therefore reads the tabulated values from the file ciexyz31_1.csv, which provides $\bar{x}, \bar{y}, \bar{z}$ at $1\ nm$ intervals between 360 and $830\ nm$ in the CIE 1931 standard [26]. The code loads this table and interpolates it onto the working wavelength grid using linear interpolation, after which the XYZ integration is performed as a matrix vector product between the interpolated sensitivity functions and the per wavelength sin^2 intensities. A final auto exposure step normalizes the RGB array by its peak value before gamma correction, simulating the brightness adjustment an experimenter would make on a real microscope to make optimal use of the display's dynamic range, and ensuring that higher order colors, which are intrinsically dimmer due to partial spectral mixing, appear at a comparable brightness to first order colors rather than vanishing into the background.

VI. References

(1) Baratta, I. A.; Dean, J. P.; Dokken, J. S.; Hale, J. S.; Richardson, C. N.; Rognes, M. E.; Scroggs, M. W.; Sime, N.; Wells, G. N. *DOLFINx: The next Generation FEniCS Problem Solving Environment*; 2023; Vol. 1. https://doi.org/10.5281/zenodo.10447666 (accessed 2026-03-16).

(2) Scroggs, M. W.; Dokken, J. S.; Richardson, C. N.; Wells, G. N. Construction of Arbitrary Order Finite Element Degree-of-Freedom Maps on Polygonal and Polyhedral Cell Meshes. *ACM Transactions on Mathematical Software* **2022**, *48* (2). https://doi.org/10.1145/3524456.

(3) Scroggs, M. W.; Baratta, I. A.; Richardson, C. N.; Wells, G. N. Basix: A Runtime Finite Element Basis Evaluation Library. *J. Open Source Softw.* **2022**, *7* (73). https://doi.org/10.21105/joss.03982.

(4) Alnæs, M. S.; Logg, A.; Ølgaard, K. B.; Rognes, M. E.; Wells, G. N. Unified Form Language: A Domain-Specific Language for Weak Formulations of Partial Differential Equations. *ACM Transactions on Mathematical Software* **2014**, *40* (2). https://doi.org/10.1145/2566630.

(5) Balay, S.; Abhyankar, S.; Adams, M.; Brown, J.; Brune, P.; Buschelman, K.; Constantinescu, E.; Dalcin, L.; Benson, S.; Dener, A.; Eijkhout, V.; Faibussowitsch, J.; Gropp, W.; Hapla, V.; Isaac, T.; Jolivet, P.; Karpeev, D.; Kaushik, D.; Knepley, M.; Kong, F.; Kruger, S.; May, D.; McInnes, L.; Mills, R.; Mitchell, L.; Munson, T.; Roman, J.; Rupp, K.; Sanan, P.; Sarich, J.; Smith, B.; Suh, H.; Zampini, S.; Zhang, H.; Zhang, J. *PETSc/TAO Users Manual Revision 3.24*; 2025. https://doi.org/10.2172/2998643.

(6) Dalcin, L. D.; Paz, R. R.; Kler, P. A.; Cosimo, A. Parallel Distributed Computing Using Python. *Adv. Water Resour.* **2011**, *34* (9). https://doi.org/10.1016/j.advwatres.2011.04.013.

(7) Butcher, J. C. *Numerical Methods for Ordinary Differential Equations*; 2016. https://doi.org/10.1002/9781119121534.

(8) Amestoy, P. R.; Duff, I. S.; L'Excellent, J. Y.; Koster, J. A Fully Asynchronous Multifrontal Solver Using Distributed Dynamic Scheduling. *SIAM Journal on Matrix Analysis and Applications* **2002**, *23* (1). https://doi.org/10.1137/S0895479899358194.

(9) Amestoy, P. R.; Buttari, A.; L'Excellent, J. Y.; Mary, T. Performance and Scalability of the Block Low-Rank Multifrontal Factorization on Multicore Architectures. *ACM Transactions on Mathematical Software* **2019**, *45* (1). https://doi.org/10.1145/3242094.

(10) Siddiquee, Z.; Talwar, M.; Jákli, A. FNLC Bifocal Microlenses. Zenodo May 2026. https://doi.org/10.5281/zenodo.20127570.

(11) W Oseen, B. C. THE THEORY OF LIQUID CRYSTALS. *Transactions of the Faraday Society* **1933**, *29* (140), 883–899.

(12) Frank, F. C. I. Liquid Crystals. On the Theory of Liquid Crystals. *Discuss. Faraday Soc.* **1958**, *25* (I), 19. https://doi.org/10.1039/df9582500019.

(13) Vaupotič, N.; Pociecha, D.; Rybak, P.; Matraszek, J.; Čepič, M.; Wolska, J. M.; Gorecka, E. Dielectric Response of a Ferroelectric Nematic Liquid Crystalline Phase in Thin Cells. *Liq. Cryst.* **2023**, *50* (4), 584–595. https://doi.org/10.1080/02678292.2023.2180099.

(14) Meyer, R. B. Piezoelectric Effects in Liquid Crystals. *Phys. Rev. Lett.* **1969**, *22* (18), 918–921.

(15) Tóth-Katona, T.; Jánossy, I.; Salamon, P.; Jákli, A. Light-Induced Thermomechanical Reorientation and Helicity Flipping in Ferroelectric Nematic Phase. *Commun. Mater.* **2025**, *6* (1). https://doi.org/10.1038/s43246-025-00948-3.

(16) Nishikawa, H.; Shiroshita, K.; Higuchi, H.; Okumura, Y.; Haseba, Y.; Yamamoto, S. I.; Sago, K.; Kikuchi, H. A Fluid Liquid-Crystal Material with Highly Polar Order. *Advanced Materials* **2017**, *29* (43), 1702354. https://doi.org/10.1002/adma.201702354.

(17) Chen, X.; Martinez, V.; Korblova, E.; Freychet, G.; Zhernenkov, M.; Glaser, M. A.; Wang, C.; Zhu, C.; Radzihovsky, L.; Maclennan, J. E.; Walba, D. M.; Clark, N. A. The Smectic ZA Phase: Antiferroelectric Smectic Order as a Prelude to the Ferroelectric Nematic. *Proc. Natl. Acad. Sci. U. S. A.* **2023**, *120* (8), e2210062119. https://doi.org/10.1073/pnas.2217150120.

(18) de Gennes, P. G.; Prost, J. *The Physics of Liquid Crystals*, 2nd ed.; Claredon Press: Oxford, 1993.

(19) Ghimire, A.; Basnet, B.; Wang, H.; Guragain, P.; Baldwin, A.; Twieg, R.; Lavrentovich, O. D.; Gleeson, J.; Jakli, A.; Sprunt, S. Dynamics of the Antiferroelectric Smectic-ZAphase in a Ferroelectric Nematic Liquid Crystal. *Soft Matter* **2025**, *21* (44), 8510–8522. https://doi.org/10.1039/d5sm00796h.

(20) Rapini, A.; Papoular, M. Distortion D’une Lamelle Nématique Sous Champ Magnetique Conditions D’ancarage Aux Parois. *Le Journal de Physique Colloques* **1969**, *30* (C4), 54–56. https://doi.org/10.1051/jphyscol:1969413.

(21) Kumari, P.; Basnet, B.; Lavrentovich, M. O.; Lavrentovich, O. D. Chiral Ground States of Ferroelectric Liquid Crystals. *Science (1979).* **2024**, *383*, 1364–1368.

(22) Grönfors, E.; Rudquist, P. Elastic Deformations in Ferroelectric Nematic Liquid Crystals Analyzed in Terms of a Large Effective Splay Elastic Constant. *PREPRINT (version 1) available at Research Square* **2026**. https://doi.org/10.21203/rs.3.rs-8395646/v1.

(23) Sakhawat, M.; Himel, H.; Gleeson, J. T.; Twieg, R. J.; Sprunt, S.; Jákli, A. Electrochromic Chiral Ferroelectric Nematic Liquid Crystals. *ArXiv 2603.04641v1*, 1–20.

(24) Paul, A.; Paul, M.; Badu, M.; Ghimire, A.; Dhakal, N. P.; Sprunt, S.; Jákli, A.; Gleeson, J. T. Comprehensive Characterization of a Reference Ferroelectric Nematic Liquid Crystal Material. *Materials* **2025**, *18*, 5496–5509.

(25) Fairman, H. S.; Brill, M. H.; Hemmendinger, H. How the CIE 1931 Color-Matching Functions Were Derived from Wright-Guild Data. *Color Res. Appl.* **1997**, *22* (1). https://doi.org/10.1002/(SICI)1520-6378(199702)22:1<11::AID-COL4>3.0.CO;2-7.

(26) Schanda, J. *Colorimetry: Understanding the CIE System*; 2007. https://doi.org/10.1002/9780470175637.
